# Application of the FFLUX Force Field to Molecular Crystals: A Study of Formamide

**DOI:** 10.1021/acs.jctc.3c00578

**Published:** 2023-10-17

**Authors:** Matthew L. Brown, Jonathan M. Skelton, Paul L. A. Popelier

**Affiliations:** Department of Chemistry, The University of Manchester, Oxford Road, Manchester M13 9PL, Britain

## Abstract

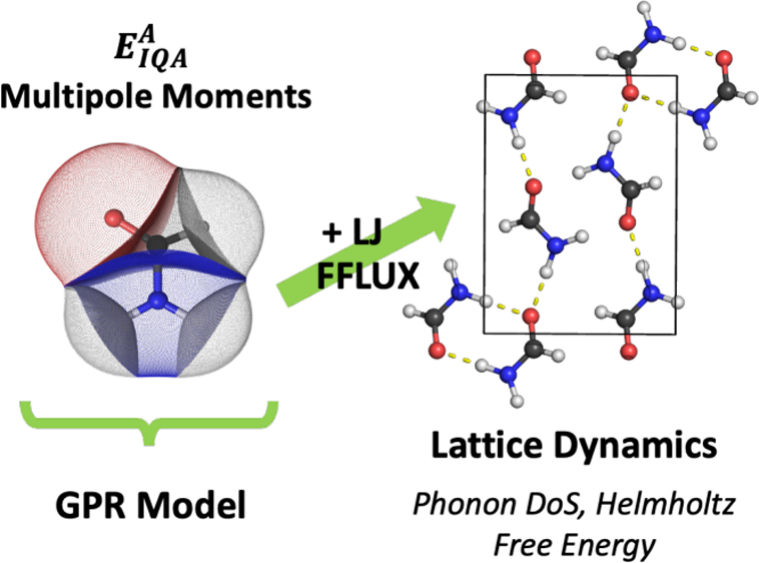

In this work, we present the first application of the quantum chemical topology force field FFLUX to the solid state. FFLUX utilizes Gaussian process regression machine learning models trained on data from the interacting quantum atom partitioning scheme to predict atomic energies and flexible multipole moments that change with geometry. Here, the ambient (α) and high-pressure (β) polymorphs of formamide are used as test systems and optimized using FFLUX. Optimizing the structures with increasing multipolar ranks indicates that the lattice parameters of the α phase differ by less than 5% to the experimental structure when multipole moments up to the quadrupole are used. These differences are found to be in line with the dispersion-corrected density functional theory. Lattice dynamics calculations are also found to be possible using FFLUX, yielding harmonic phonon spectra comparable to dispersion-corrected DFT while enabling larger supercells to be considered than is typically possible with first-principles calculations. These promising results indicate that FFLUX can be used to accurately determine properties of molecular solids that are difficult to access using DFT, including the structural dynamics, free energies, and properties at finite temperature.

## 1 Introduction

The structure of molecular crystals is known to be strongly linked to physical properties such as color,^1^ bioavailability,^2^ and solubility.^3^ The flexibility of molecular species typically results in polymorphism, where molecules can form multiple crystal structures that can and do differ in physical properties. Hence, being able to accurately predict crystal structures is considered an important challenge in many areas of industry.

Computational methods for predicting the structure of molecular crystals have shown significant improvement in the past two decades, as highlighted by blind tests of organic crystal structure prediction (CSP) methods.^4−9^ CSP is fast becoming a useful tool for understanding the crystal energy landscapes of molecular solids and complementing experimental polymorph screening.^10−12^

While force fields have been used to study crystal structures for many years,^13−16^ in the most recent blind tests, periodic dispersion-corrected density functional theory (DFT+D) calculations have become increasingly prevalent. These methods allow for more accurate lattice energy and free energy calculations than can typically be obtained with standard force fields.

The inadequacy of traditional force fields can be put down to the parametrization of the potential energy surface (PES) into a series of approximate potentials that can lead to significant errors in energy and force calculations. This is often compounded by the use of point charges to represent electrostatics despite the demonstrable improvements in accuracy from higher-order multipole moments (see ref (17) for a provocative perspective). The combination of these issues means that traditional force fields are often not able^18^ to accurately calculate the relative energy differences between polymorphs, which are typically only a few kJ mol^–1^. To circumvent this issue, DMACRYS^19^ uses multipole moments up to the hexadecapole moment but is restricted to a rigid-body representation of the component molecules.

Despite the improvements seen with DFT+D methods over force fields, recent studies have shown that the delocalization error present in common semilocal functionals can limit the accuracy of the lattice energy ranking. One notable example of this shortcoming is the exaggeration of the difference in energy between stable planar forms of π-conjugated systems compared to competing nonplanar forms.^20^ This effect was shown in a study of the molecule 5-methyl-2-[(2-nitrophenyl)amino]-3-thiophenecarbonitrile, nicknamed “ROY” due to its red, orange, and yellow polymorphs. Here, the incorrect ranking of the red and orange polymorphs containing more planar molecules as the most stable structures (relative to the yellow polymorphs) was primarily attributed to the delocalization error.^12^ Replacing the intramolecular energy with a more expensive wave function-based method allowed for the correct ranking of structures in this case. However, the use of these methods is not always technically feasible.

FFLUX^21,22^ is a new force field that has previously been used to accurately predict the properties of liquid water^23^ and the geometries of gaseous formamide dimers.^24^ The dimer work showed that FFLUX effectively “sees” the electrons, with small changes in C=O and C–N bond lengths due to hydrogen bonding captured accurately relative to the training level of theory. The correct energy ranking of the dimeric minima was also obtained, suggesting that an accurate ranking of crystal polymorphs could be possible. FFLUX utilizes Gaussian process regression^25^ (GPR) models trained on data from the interacting quantum atom (IQA) energy partitioning scheme^26^ to predict an intramolecular PES that lies closer to quantum mechanics than traditional force fields. Models of the atomic multipole moments also allow for the prediction of moments up to the hexadecapole moment that change with the geometry of a molecule, which means that FFLUX allows the restriction to rigid body molecules to be lifted while retaining the accuracy of higher order multipole moments.

AMOEBA is another multipolar force field that allows for flexible molecules with a multipolar representation of electrostatics. AMOEBA uses permanent multipole moments up to the quadrupole and induced dipole moments.^27,28^ The approach taken in FFLUX differs significantly from that taken in AMOEBA. In FFLUX, multipole moments (up to the hexadecapole) respond to a change in the geometry of the molecule. This is possible in AMOEBA+(CF)^29^ but currently only with charges. In FFLUX, intramolecular polarization is captured naturally by the geometry-dependent moments, while in AMOEBA, the induced dipole moments and the use of a Thole-style damping function allow for intra- and intermolecular polarization. In previous work with FFLUX, GPR models have been trained using an implicit solvent to approximate intermolecular polarization,^23^ but in principle, intermolecular polarization can also be captured using models trained on clusters of the molecule at hand. AMOEBA has previously been used in simulations of organic crystals^30,31^ and liquid water,^29^ both of which are target systems for FFLUX.

The GPR models trained for FFLUX calculations are capable of sub-kJ mol^–1^ accuracy, making them potentially useful for studying molecular crystals, where such accuracy is often required to capture the small energy differences between polymorphs. Moreover, models can be trained using wave function methods, which could eliminate the delocalization error seen in common functionals while mitigating the computational expense of these higher-level methods and therefore allowing for geometry optimizations and dynamics simulations that would otherwise not be feasible.

**Figure 1:**
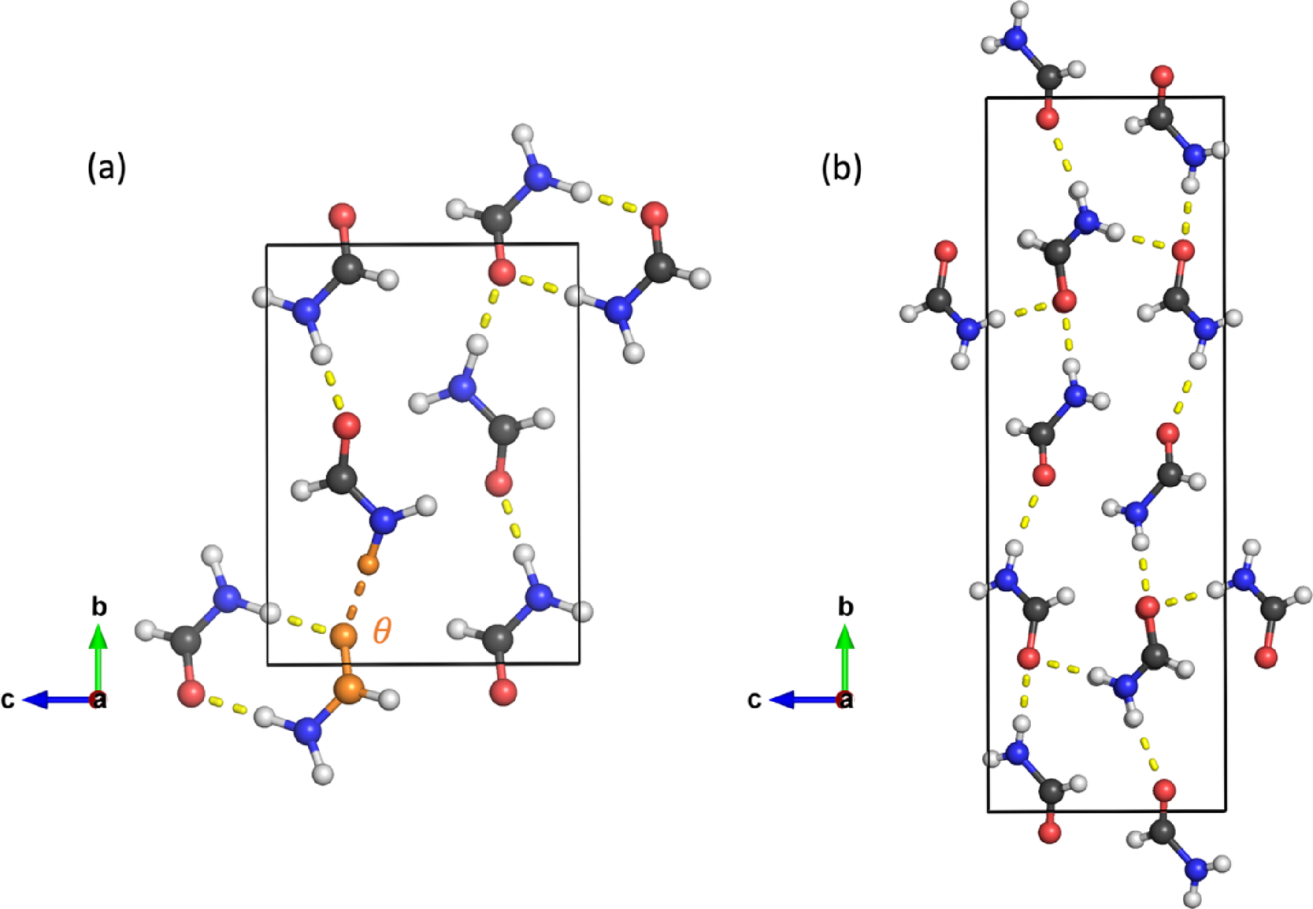
Unit cells of the (a) ambient-pressure α phase containing 4 molecules and (b) high-pressure β phase of formamide containing 8 molecules. The red, green, and blue axes show the *a*, *b*, and *c* directions, respectively, and the atom colors are as follows: H, white; C, black; N, blue; O, red. The inter-ring angle, θ, depicted in orange has been seen to open up significantly in previous force field optimizations.

As a proof of concept for FFLUX’s use in solid-state calculations, we apply it to formamide crystals, chosen due to the molecule’s small size and the existence of two polymorphs (Figure 1), allowing the relative energies to be studied. Previous force field optimizations of α formamide^32,33^ have encountered a variety of issues including the “inter-ring” C=O···H angle opening up from 129° to 141°, which also makes this system an example of a case where traditional force fields can fail. This inter-ring angle is indicated in orange and labeled θ in Figure 1. Note that the term “ring” refers to a doubly hydrogen-bonded dimer, which is visible in the bottom-left corner of Figure 1a.

In this work, the FFLUX force field is used in solid-state calculations for the first time. Geometry optimization of both α and β phases of formamide is performed using FFLUX and compared to dispersion-corrected PBE+D3. The unit cell and molecular geometry are compared with the experimental geometries. Lattice dynamics calculations are also performed to obtain the density of states (DoS) within the harmonic approximation using the Phonopy package,^34^ and extension to the quasi-harmonic approximation is shown to be feasible.

## 2 Methods

### 2.1 The FFLUX Force Field

The FFLUX force field is based on the principles of quantum chemical topology (QCT), a family of methods that share the idea of a (gradient) vector field partitioning a quantum mechanical function.

QCT uses the language (e.g., separatrix, basin, critical point, and attractor) of a mathematical area called dynamic system theory. Data from the IQA partitioning scheme, which itself is an extension of the quantum theory of atoms in molecules (QTAIM),^35^ are used to train GPR models. These models allow for prediction of atomic energies that can then be used to accurately compute intramolecular energies as well as multipole moments that can be used to evaluate intermolecular electrostatics. These models allow FFLUX simulations to yield results closer to quantum mechanics than is possible with the parametrizations used in traditional force fields. The FFLUX force field is implemented in the in-house DL_FFLUX code, built on the simulation program^36^ DL_POLY 4, which therefore offers DL_FFLUX access to DL_POLY routines including geometry optimizers and various numerical integrators for molecular dynamics simulations.

#### 2.1.1 The Quantum Theory of Atoms in Molecules

At the heart of FFLUX are objects called topological atoms. Topological atoms are found by the QTAIM partitioning,^35^ where a gradient vector field is applied to the electron density of a molecule or group of molecules. Doing this produces trajectories of gradient vectors that move from infinity to critical points in the electron density. These trajectories are named gradient paths and “carve out” the topological atoms from the electron density. Each topological atom is made up of a collection of gradient paths that move toward a maximum in the electron density (chemically speaking, a nucleus). The boundaries between atoms are defined by a series of gradient paths that terminate at a saddle point (one of two possible types) in the density (bond critical point), forming a zero-flux surface or an interatomic surface (IAS). These paths obey the equation
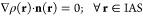
1where **n**(**r**) is a normal vector to the surface at point **r**.

**Figure 2:**
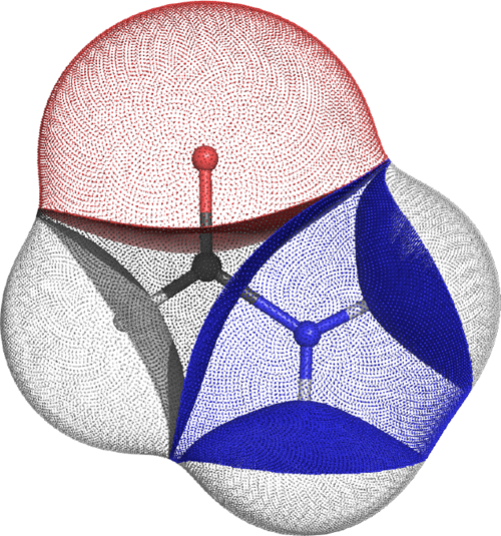
Formamide molecule partitioned into its constituent topological atoms. This image has been prepared with the in-house code PyMol-QTAIM Visualizer, written by F. Falcioni and M. J. Burn.

Topological atoms are obtained without the use of a reference density with all information for the partitioning coming from the molecular electron density itself. These atoms are also space-filling and non-overlapping by construction. An example of a partitioned formamide molecule is shown in Figure 2.

#### 2.1.2 The Interacting Quantum Atom Partitioning Scheme

Based on the partitioning defined by QTAIM, IQA^26^ rigorously partitions the one- and two-particle density matrices to obtain atomic energies that recover the wave function energy when summed. IQA is a general and rigorous partitioning scheme,^37^ producing chemically meaningful energetic terms. IQA has previously been used to study a variety of phenomena including hydrogen bonding^38,39^ and aromaticity.^40^ IQA has also been applied to proteins like HIV-1 protease where peptide hydrolysis was studied^41^ and the known polymorphs of succinic acid^42^ using a central molecule surrounded by neighboring molecules to mimic the crystalline environment. Recently, the partitioning has also been extended to periodic solids^43^ and applied to allotropes of carbon and polymorphs of boron nitride as well as crystals of a variety of small molecules.

The energy of a topological atom, , can be decomposed into intra- and interatomic contributions as shown in eq 2,
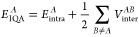
2where  and  are the intra- and interatomic energies, respectively, of a topological atom *A*. These two terms can be further partitioned into additional energy terms, again calculated as integrations over the volumes of the topological atoms, but these terms are not currently relevant for the GPR models used within FFLUX. The  term contains the “classical” electrostatic energy, which can be approximated by the well-known multipolar expansion (to a very high degree of accuracy except when diverging). It should be clear that long-range electrostatics are calculated by inserting into this expansion atomic multipole moments, which are themselves calculated by the aforementioned volume integration. Note that atomic energies and multipole moments are both obtained from the same universal, topological integration scheme. We believe that this consistency is important in the construction of a future-proof force field.

#### 2.1.3 Gaussian Process Regression

The GPR models used in FFLUX calculations capture short-range (intramolecular) interactions by using models trained on atomic energies. Each atom that is being modeled has its own GPR model, with the model having “knowledge” of its surroundings. The prediction of a property *Q* made by a model  therefore depends on all the atoms in the system, not just the atom *A* that the model is trained for, where in this context, the system can be a single molecule or a larger multimolecule entity such as a dimer.

In simulations, interactions between molecules are modeled based on electrostatic and van der Waals interactions, with the electrostatic interactions accounted for using the multipole moments predicted by the GPR models. Much like each atom in the system has its own model trained on IQA energies, each atom also has a series of models, one model trained on each of its multipole moments. The GPR models thus allow for flexible multipole moments that depend on the atomic environment and introduce additional force terms into the electrostatics. A modified version of the smooth particle mesh Ewald (SPME) method^44^ is used to account for this fact. The derivation of these terms is given in ref (45). So, the FFLUX method allows one to break free from the typical rigid-body constraint of multipolar electrostatics.

In this work, we utilize a monomeric model, where the GPR models are trained on a single formamide molecule. Van der Waals interactions are therefore not machine-learned in the way that electrostatics are. Instead, the calculations presented here use a traditional Lennard-Jones potential. However, it is also possible to utilize *N*-meric modeling where the GPR models then also predict intermolecular interactions after having been trained on N-mers. This future capability will soon eliminate the need for nonbonded potential energy functions. This important extension is more challenging to the machine learning engine and is still under development in our lab.

In GPR, the covariance between two points is calculated by using a covariance kernel. In this study, we use a modified radial basis function (RBF) kernel, which takes into account that every third feature is an angular feature ranging from −π to +π in value. This kernel is shown in eq 3,
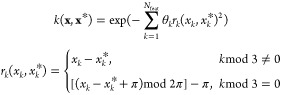
3

The hyperparameters θ_*k*_ scale the distance between the *k* features of the training points (**x** and x*) and are optimized for the training set by maximizing a log likelihood function. To maintain a dimensionless exponential, this parameter has units that are the reciprocal of the corresponding feature.

Features in the models trained for the FFLUX simulations are defined in a series of atomic local frames (ALFs). The origin of each ALF is the atom for which the model is being trained (atom *A*). Two atoms are required to fix the *x*-axis (atom *A*_*x*_) and the *xy*-plane (atom *A*_*xy*_). These atoms are determined by the Cahn–Ingold–Prelog rules, with the highest and second highest priority atoms being assigned to *A*_*x*_ and *A*_*xy*_, respectively. The *z*-axis is then constructed orthogonally to form a right-handed axis system. The first three features are the distance between *A* and *A*_*x*_, the distance between *A* and *A*_*xy*_, and the *A*_*x*_ – *A*– *A*_*xy*_ angle. All other atoms are subsequently described by spherical coordinates relative to the ALF. Each model therefore has 3*N* – 6 features, where *N* is the number of atoms. Hence, the formamide model is 12-dimensional.

Predictions are made using the GPR models according to eq 4,
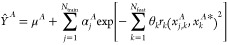
4Here, *Ŷ*^*A*^ is the predicted energy or multipole moment of atom *A*, μ^*A*^ is the average value of the output over all the training points,  is the weight of the *j*-th training point,  is the *k-*th feature of the *j-*th training point, and  is the *k-*th feature of the unseen point. The function *r*_*k*_ obeys the conditions shown in eq 3.

### 2.2 Lattice Dynamics

The lattice vibrations (phonons) in solids can be used to model the natural thermal motion at finite temperature and to predict how the physical properties of crystals vary with temperature. These calculations can also provide access to a variety of experimentally relevant quantities such as vibrational spectra.^46^ At the most basic level, phonons can be modeled within the harmonic approximation using the framework of lattice dynamics.

Within the harmonic approximation, the second-order force-constant matrices, ϕ_αβ_, are calculated as
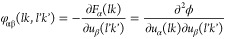
5where φ is the potential energy of the crystal, *u*(*lk*) is the displacement of the *k-*th atom in the *l-*th unit cell from its equilibrium position, and *F*_α_(*lk*) is the corresponding force, with the subscripts α and β referring to the Cartesian directions.

Applying Bloch’s theorem yields the dynamical matrix, **D**(**q**),
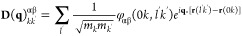
6where **r**(*kl*) is the position of the *k-*th atom in the *l-*th unit cell, with mass *m*_*k*_, and **q** is the phonon wavevector defined in the reciprocal space (Brillouin zone) of the crystal. For a crystal with *n*_*a*_ atoms in the primitive unit cell, diagonalizing this matrix gives the 3*n*_*a*_ phonon frequencies denoted ω(**q**, *j*) and their corresponding displacement vectors **W**(**q**, *j*) at the phonon wavevector **q** with band index *j*.^34^ Computing the phonon frequencies on a uniform grid of **q** allows the computation of the phonon density of states, *g*(ω), showing the number of modes as a function of the frequency over the entire Brillouin zone,
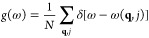
7where *N* is the number of wavevectors **q** included in the summation. The phonon frequencies can also be used to determine the thermodynamic partition function, *Q*,
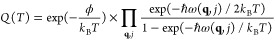
8From this equation, the constant-volume (Helmholtz) free energy, *F*, can be derived via the bridge relation
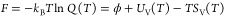
9where *U*_V_ is the vibrational internal energy and *S*_V_ is the vibrational entropy. Both vibrational terms are temperature-dependent, whereas the crystal potential energy ϕ, which can be equated to the lattice internal energy *U*_latt_, is assumed to be temperature independent.

## 3 Computational Details

### 3.1 Crystal Structures

Crystal structures of α^47^ and β^48^ formamide were obtained from the Cambridge Structural Database (CSD, Table 1).

**Table 1:** Details of the Formamide Structures Examined in This Work, Including the Temperature and Pressure at Which the Structures Were Collected and Their CSD Refcodes

phase	method	temperature/K	pressure/GPa	CSD refcode
α	X-ray diffraction	223	ambient	FORMAM^47^
β	X-ray diffraction	296	1.20	FORMAM04^48^

### 3.2 FFLUX Simulations

#### 3.2.1 GPR Models

The GPR models for the FFLUX simulations were trained using our in-house Python pipeline, ICHOR.^49^ Details of the creation of the 1506-point formamide GPR model and a test of its accuracy in monomer and dimer calculations are presented in ref (24), but a brief overview is provided here. A 1 ns AMBER simulation of a formamide monomer at 300 K was performed to generate a set of geometries. The trajectory from this simulation was then split into three sets of geometries: (i) a training set on which the model was trained (initially 36 points), (ii) a validation set of 500 randomly selected points to test the model, and (iii) a sample set of 100,000 randomly selected points. Wave functions for the training points were then calculated in GAUSSIAN09,^50^ and an IQA analysis was performed using AIMAll^51^ to obtain atomic energies and multipole moments. Geometries from the sample set were added to the training set to iteratively improve the model, with the “best” points chosen using adaptive sampling,^52^ until the model was deemed to be of a suitable quality. In this case, the maximum error across the 500-point validation set was 0.8 kJ mol^–1^, and 50% of the predictions had errors of less than 0.07 kJ mol^–1^. Within the ICHOR pipeline, the in-house program FEREBUS^53^ is used to train the GPR models on the atomic energy and multipole data generated in previous stages.

#### 3.2.2 Intermolecular Electrostatics

FFLUX simulations can be run at different multipolar interaction ranks, represented by the quantity *L*′, which denotes the highest-ranking multipole moment present in the simulation. For example, *L*′ = 0 simulations consider only atomic charges (*l* = 0) and charge–charge interactions. *L*′ = 1 considers both charges and dipole moments (*l* = 1), and the simulations include charge–charge, charge-dipole, and dipole–dipole interactions. A given value of *L*′ essentially includes all of the possible interactions between multipole moments up to the rank specified by *L*′ in a square matrix. Note that this way of organizing the level of multipolar interaction is different to the one reported in our earlier literature and that of others. It is also possible to group interactions by the interaction rank *L* instead of *L*′. The interaction rank *L* is defined as *L* = *l*_*A*_ + *l*_*B*_ + 1, where *l*_*A*_ and *l*_*B*_ refer to the ranks of respectively atoms *A* and *B*, as illustrated above. Instead of a square matrix, one obtains a triangular matrix.

In our previous work, tests were also performed on a validation set of dimers at different *L*′ ranks, where the intermolecular atom–atom electrostatic interactions were evaluated. Multipole moments from AIMAll were used to calculate electrostatic energies that were considered the “truth”. The GPR model was then allowed to predict multipole moments to calculate the electrostatic energies for the same set of dimers. The root-mean-square error (RMSE) was then calculated and found to be, at most, 0.5 kJ mol^–1^, with higher *L*′ calculations having the largest RMSEs but the charge–charge interactions contributing the most to the error.

#### 3.2.3 Nonbonded Parameters

Monomeric GPR models, such as the formamide model used here, only “know” about the system for which they are trained and can only predict atomic energies and multipole moments. This means that molecules in a simulation can only interact electrostatically, and dispersion and repulsion have to be introduced through a nonbonded potential as in traditional force fields. For this work, a 12-6 Lennard-Jones potential is used,
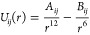
10Following the protocol presented by Meuwly et al.,^54^ the nonbonded parameters were optimized for *L*′ = 2 simulations (multipolar interactions up to quadrupole–quadrupole) by scaling the *A* and *B* parameters by a factor *n* such that
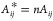
11
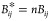
12The nonbonded parameter set suggested by Hagler et al.^32,33^ was used as a starting point, as it had previously worked as a good starting point for dimer simulations. Optimizations of α formamide were performed as described in Section 3.2.4 for each of the series of scaled parameter sets with *n* from 0.9 to 1.6. The crystal density (ρ), lattice energy (*U*_latt_), and β angle were used as properties to compare to experiment. *U*_latt_ was calculated as^55^
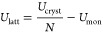
13where *U*_cryst_ is the total energy of the supercell, *N* is the number of molecules in the supercell, and *U*_mon_ is the energy of an isolated (gas-phase) monomer in its most stable conformation.

A score, *S*, was calculated for each parameter set as a weighted sum of square differences between the experimental and calculated properties:
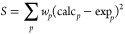
14with *w*_*p*_ = 3,  = 1, and *w*_β_ = 5. The lowest weight was assigned to the lattice energy due to the magnitude of the difference being the largest, while the largest weight was assigned to the β angle. These weights were used despite the magnitudes of the differences in the angles being larger than those in the densities, hence putting greater importance on getting the angle closer to experiment. The parameter set with the lowest score has the best match to experiment.

The chosen parameter set was that with the Hagler parameters increased by 40% (i.e., *n* = 1.4) resulting in the parameters of Table 2. Further details on the optimization of the Lennard-Jones parameters are given in Section 1 of the Supporting Information, with the initial parameters given in Table S1.1 and the calculated properties and scores obtained with each parameter set listed in Table S1.2.

**Table 2:** Nonbonded Parameters Used in the FFLUX Optimizations of Formamide Crystals

atom	*A*/kJ mol^–1^ Å^12^	*B*/kJ mol^–1^ Å^6^
C	17,701,667.200	7849.184
N	13,302,609.600	7204.848
O	1,610,840.000	2940.515

#### 3.2.4 Crystal-Structure Optimizations

FFLUX optimizations were performed in 7 × 3 × 4 and 7 × 2 × 4 supercells of the α and β phases, respectively, with 2016 and 2688 atoms, giving simulation cells with a volume of approximately (25^3^) Å^3^ and allowing for the electrostatic and nonbonded cut-offs to be set to a generous 12 Å.

Optimizations were carried out in three stages. Each stage utilized the DL_POLY 0 K optimizer, which is equivalent to performing a molecular-dynamics simulation at low temperature where, at each timestep, the atoms move in the direction of the computed forces but are not allowed to gain a velocity larger than that corresponding to a temperature of 10 K. In the first stage, a 3000-step run with a timestep Δ*t* = 1 fs was performed in the *N*σ*T* ensemble, which allows the cell lengths/angles and the atomic positions to change. In the second stage, the final geometry from the first stage was then used as the starting point for a 3000-step run in the *NVT* ensemble, in which the cell shape was fixed, but the atomic positions were allowed to continue to change. Finally, the third stage completes the optimization with another 3000-step optimization in the *N*σ*T* ensemble.

Convergence was determined by monitoring the unit-cell parameters (*a*, *b*, *c*, α, β, and γ) and the lattice energy. For the structural optimization to be considered converged, each of these seven properties had to pass two criteria. The first was that the gradient between the final (*N-*th) step and the *N* – 1000-th step had to be less than 10^–4^ kJ mol^–1^ timestep^–1^ for the lattice energy and 10^–3^ Å timestep^–1^ or deg timestep^–1^ for cell lengths and angles, respectively. The second criterion was that in each property, the RMSE deviation from the straight line connecting the *N*th and *N −*1000-th steps over the last 1000 timesteps had to be less than 0.1 units. Hence, there were no significant fluctuations in each property between the two points either. In practice, across all the optimizations performed during the study, we observed maximum fluctuations of 0.04 kJ mol^–1^, 0.12 Å, and 0.3° in the total energy, lattice lengths, and cell angles over the last 1000 steps, equating to changes of 0.06, 0.5, and 0.3%, respectively.

For the α phase, optimizations were carried out at *L*′ = 0, 1, and 2, while optimizations of the β phase were only carried out at *L*′ = 2 as this was the best performing *L*′ for the α phase. The parameters for the SPME were set using the DL_POLY directive “spme precision *f*”, which automatically optimizes SPME parameters for the precision *f*, here set to 10^–8^. A Berendsen thermostat and barostat with relaxation times of 0.1 and 0.5 ps, respectively, were used in the optimizations to control the temperature and pressure. For phonon calculations, single-point force calculations were performed with the same parameters.

A high-pressure optimization was also performed on the β phase of formamide with the external hydrostatic pressure of 1.2 GPa at which the experimental structure was collected (Table 1). DL_POLY requires that the nonbonded and electrostatic cut-offs be less than or equal to half the smallest cell dimension. To prevent premature termination of the optimization, the cut-offs were reduced to 11 Å for these calculations.

### 3.3 DFT Calculations

DFT optimizations and phonon calculations were carried out using the Vienna *Ab initio* Simulation Package (VASP) code.^56^ Electron exchange and correlation were modeled using the PBE^57^ functional, either “bare” or with the Grimme D3 dispersion correction (i.e., PBE+D3).^58^

The crystal structures from the CSD were optimized to a tolerance of 10^–2^ eV Å^–1^ on the forces, with a plane wave cut-off energy of 850 eV and the Γ-centered **k**-point sampling meshes^59^ in Table 3. Both the cut-off and **k**-point mesh were chosen to converge the absolute total energies and pressures to less than 1 meV atom^–1^ and 1 kbar (0.1 GPa), respectively. Core electrons were modeled using projector augmented-wave (PAW) pseudopotentials^60,61^ with H 1s and N, C, and O 2s/2p electrons in the valence shells.

**Table 3:** Summary of the Technical Parameters Used for the Geometry Optimizations and Phonon Calculations on α and β Formamide

		**k**-point sampling		
phase	phonon SC (no. of atoms)	optimization	phonon	cut-off/eV
α	4 × 2 × 2 (384)	6 × 2 × 3	2 × 1 × 2	850
β	4 × 1 × 2 (384)	3 × 1 × 1	1 × 1 × 1	850

### 3.4 Lattice Dynamics

Lattice dynamics calculations were performed on the structures optimized with PBE+D3 and FFLUX using the Phonopy package, which was used to set up and postprocess supercell finite-displacement phonon calculations. The supercell expansions of the PBE+D3 optimized structures given in Table 3 were used for the DFT Phonopy calculations, while the FFLUX Phonopy calculations were performed using the optimized supercells. For the PBE+D3 calculations, the supercell single-point force calculations were performed in VASP with the reduced **k**-point meshes listed in Table 3. The FFLUX single-point calculations were performed by using the simulation settings described in Section 3.2.4. The phonon DoS was evaluated by interpolating the phonon frequencies onto regular 16 × 16 × 16 **q**-point meshes for the PBE+D3 calculations and 2 × 2 × 2 meshes for the FFLUX calculations.

## 4 Results and Discussion

### 4.1 Optimized Crystal Structures

Table 4 compares the FFLUX- and PBE+D3-optimized lattice constants of α and β formamide to the experimental parameters. For α formamide, FFLUX optimizations were performed at multiple values of *L*′ to investigate the effect of multipolar rank on the accuracy of the optimization. In general, increasing *L*′ yields lattice parameters closer to experiment such that at *L*′ = 2 the percentage differences are all within 3%. This success is evidence of the importance of multipole moments for an accurate representation of the electrostatics. In work by Ponder et al.,^30^ the α formamide crystal collected by Stevens^62^ was optimized using the AMOEBA force field. In this work, the *a*, *b*, *c*, and β lattice parameters were found to differ between 1.6 and 3.8% with the α and γ angles predicted exactly. When compared to the *L*′ = 2 FFLUX calculations (where both FFLUX and AMOEBA are using up to the quadrupole moment), FFLUX can be considered to be performing well with lattice parameters differing by, at most, 2.8%. Moreover, the differences to experiment obtained with *L*′ = 2 are of comparable magnitude to those obtained after optimization with PBE+D3, which shows that FFLUX is able to predict unit cell parameters with a similar accuracy to dispersion-corrected DFT methods.

**Table 4:** Comparison of the Predicted and Measured Unit Cell Parameters of α and β Formamide^a^

method	*a*, *b*, *c*/Å	Δ%	α, β, γ/°	Δ%
α formamide				
experiment^47^	3.69, 9.18, 6.87		90.0, 98.0, 90.0	
*L*′ = 0	3.86, 8.10, 6.65	4.6, −11.7, −3.2	90.0, 79.8, 90.0	0.0, −18.6, 0.0
*L*′ = 1	3.75, 9.59, 6.44	1.6, 4.4, −6.3	90.0, 92.1, 90.0	0.0, −6.0, 0.0
*L*′ = 2	3.71, 8.92, 6.92	0.6, −2.8, 0.7	90.0, 99.2, 90.0	0.0, 1.3, 0.0
PBE	4.27, 9.20, 6.99	15.7, 0.2, 1.7	90.0, 94.9, 90.0	0.0, −3.2, 0.0
PBE+D3	3.62, 9.12, 6.84	–1.9, −0.6, −0.4	90.0, 99.8, 90.0	0.0, 1.9, 0.0
β formamide				
experiment^48^	3.56, 18.86, 6.25		90.0, 93.8, 90.0	
*L*′ = 2	3.84, 19.11, 6.17	7.9, 1.3, −1.2	90.0, 95.2, 90.0	0.0, 1.5, 0.0
hp *L*′ = 2	3.75, 19.44, 5.82	5.3, 3.1, −6.9	90.0, 94.8, 90.0	0.0, 1.1, 0.0
PBE	4.25, 18.91, 6.95	19.3, 0.3, 11.2	90.0, 80.5, 90.0	0.0, −14.1, 0.0
hp PBE	3.63, 18.46, 6.55	2.0, −2.1, 4.8	90.0, 92.6, 90.0	0.0, −1.3, 0.0
PBE+D3	3.61, 18.58, 6.44	1.6, −1.5, 3.1	90.0, 93.5, 90.0	0.0, −0.3, 0.0
hp PBE+D3	3.44, 18.22, 6.40	–3.4, −3.4, 2.4	90.0, 95.0, 90.0	0.0, 1.3, 0.0

a FFLUX calculations were carried out at *L*′ = 0, 1, and 2, and DFT calculations were performed using PBE and PBE+D3. Percentage differences to the experimental lattice parameters are shown for comparison, where the experimental values for β formamide were obtained under a pressure of 1.2 GPa. The high-pressure optimizations on β formamide are denoted by “hp”.

While not used in this work, a representation of dispersion within the FFLUX methodology can be obtained from electron correlation energies calculated in the IQA partitioning. GPR models can be trained on the correlation energies to obtain an environment-dependent dispersion. However, intermolecular dispersion is currently provided in the form of a Lennard-Jones potential. In all cases, it is pleasing to see that the α and γ angles, which are found as 90° in the experimental structures, were well preserved in the FFLUX calculations without any imposition of symmetry. Moreover, the *P*2_1_/*c* space group of α formamide and its translational symmetry are preserved within 1 × 10^–2^ Å in the FFLUX optimizations at *L*′ = 2.

The same nonbonded parameters that were used for the α phase were again used to optimize the high-pressure β phase, but their effectiveness is questionable. Two optimizations were performed with FFLUX, one at ambient pressure and one with a constant external hydrostatic pressure of 1.2 GPa. In the ambient-pressure optimization, the *a* parameter is the worst predicted with a large expansion of 7.9% compared to the experimental structure. It is tempting to link this fact to the layering seen in the β formamide crystal. A layer consists of a hydrogen-bonded network of formamide molecules spreading out in two dimensions. These formamide molecules lie in an approximate plane that is parallel to the *b* axis, cutting off the *a* and *c* axes at one cell length (i.e., the (101) plane). These layers are stacked parallel to *b*. The stacking direction follows the direction of *a* and *c* in equal measure. It is known that in layered crystals, the bonding (or rather intermolecular cohesion) between layers is generally weaker than that within layers.^63^ As a result, when hydrostatic (nondirectional) pressure is applied, the crystal compresses more easily along the direction in which layers of molecules are stacked. When high-pressure structures are subsequently optimized under ambient pressure, the stacking direction thus tends to expand more than others, resulting in larger percentage changes. However, this argument does not explain the contraction of the *c* parameter in the FFLUX-optimized structures of β formamide compared to the experimental structure. In the FFLUX optimizations at ambient and high pressure, the *c* direction is compressed rather than expanded as should be expected. While the FFLUX optimizations do not produce the expected result compared to experimental structures, we note that the high-pressure FFLUX optimization of β formamide does predict compression along both *a* and *c* relative to the ambient pressure optimization.

We believe that the Lennard-Jones parameters optimized for α formamide are actually unsuitable for the β phase. If the molecular environments in the ambient- and high-pressure phases are significantly different, one may expect there to be significant differences in the intermolecular interactions. If this is the case, then it is likely that the fixed *A* and *B* values in the Lennard-Jones potential are unable to reproduce the dispersion and repulsive interactions for both phases accurately. In keeping with this, PBE+D3 gives reasonable results for both phases, with the *a* and *c* parameters both expanding compared to the experimental structure in the β phase. The D3 correction has geometry-dependent *C*_6_ coefficients that are adjusted based on the local geometry around the atoms. This allows for a better representation of the dispersion in different environments. The sensitivity of lattice parameters to the representation of dispersion in the simulations can be seen by comparing the PBE and PBE+D3 calculations. Bare PBE calculations generally predict significant expansions of the lattice parameters compared to the experimental structures, and inclusion of the D3 correction generally leads to a better representation of the experimental structures.

Looking to the future, it is in principle possible to mitigate the issue of “static” nonbonded parameters within the FFLUX framework using the *N*-meric modeling described previously. Unpublished work shows that replacing the nonbonded potential with machine-learned intermolecular interactions does indeed allow for more accurate simulations. However, for this technique to be applied to crystal structures will require significant changes to the DL_FFLUX code and thus represents a longer-term goal.

The RMSE between the predicted and measured α formamide structures (excluding hydrogen atoms) shows improvement with increasing *L*′, with values of 2.11, 0.86, and 0.38 Å for *L*′ = 0, 1, and 2, respectively. When optimized under ambient pressure, the β phase has an RMSE of 0.66 Å despite the poorer performance in recovering the lattice parameters. RMSEs are given in Section 2 of the Supporting Information (Table S2.1). Despite the improvement of the RMSE with *L*′, the inter-ring angle that previous force fields have struggled to capture is improved only slightly. The experimental value for the angle is 129.3°. With FFLUX, we obtain a value of 121.3° when optimizing at *L*′ = 2, whereas force field calculations from Hagler et al. obtained 141°.^33^ The FFLUX-optimized value is thus only marginally closer to experiment. Given the improved representation of electrostatics in FFLUX, the fact that the angle is still not captured well suggests that representations of dispersion and repulsion rather than of the electrostatics are at issue. This is supported by the inter-ring angles of 127.0° and 126.1° in the PBE+D3- and PBE-optimized geometries, which equate to small percentage errors of −1.8 and −2.5% relative to the experimental structure, respectively. While the difference between the two DFT methods is small, the inclusion of the dispersion correction does give an improved representation of the experimental structure. We therefore tentatively attribute the smaller than expected improvement seen with FFLUX, compared to previous force field calculations, to issues with the nonbonded parameters.

### 4.2 Energy–Volume Curves

**Figure 3:**
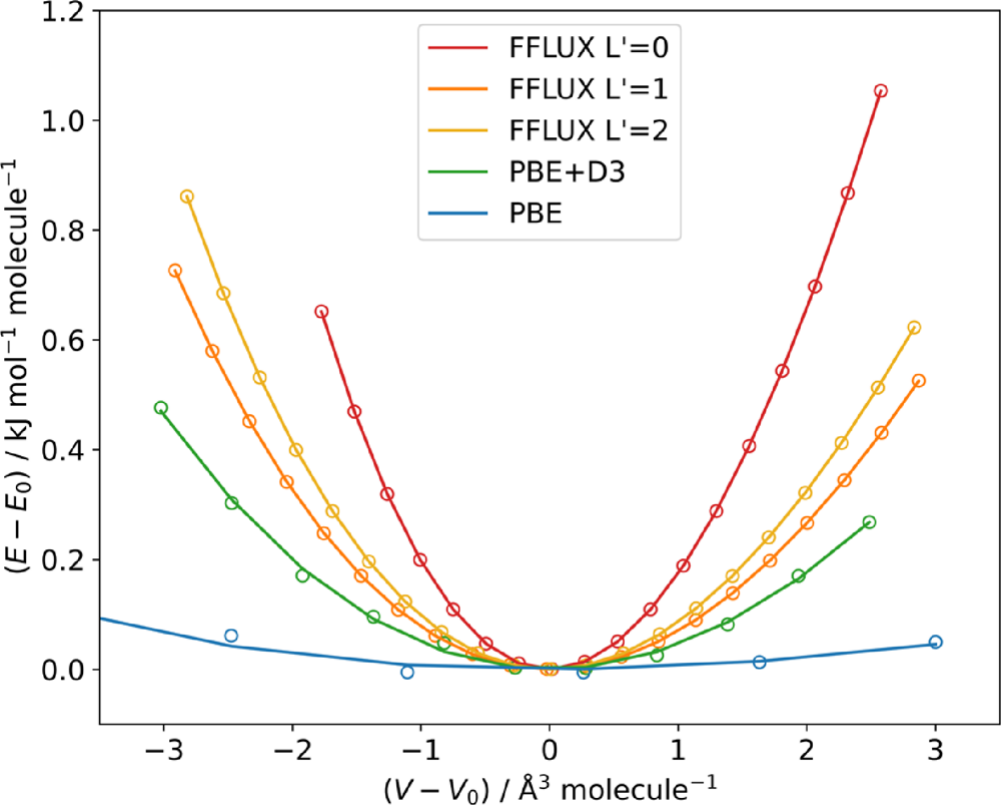
Energy–volume curves for α formamide calculated with PBE, PBE+D3, and FFLUX with *L*′ = 0, 1, or 2 over 5% expansions and compressions of the optimized volumes obtained with each method. Due to the very shallow curvature obtained with PBE, it was necessary to calculate up to 10% expansions and compressions to capture the minimum energy point, of which expansions in the range of 2–10% are shown here.

We also consider the behavior under expansion and compression of the lattice volume. Energy–volume curves are shown in Figure 3, with energies fit to the Birch–Murnaghan equation of state (EoS) given by^64^
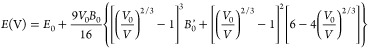
15Here, *E*_0_ and *V*_0_ are the equilibrium energy and volume, and *B*_0_ and  are the bulk modulus and its derivative with respect to pressure, respectively.

The curvature of the energy–volume curves predicted using FFLUX is steeper than that obtained with PBE+D3, which leads to significantly larger values for the bulk modulus (9.87 compared to 4.99 GPa). The volumes from each of the optimizations, *V*_opt_, are given for comparison in Table 5, and the fitted equilibrium values generally differ by <0.1%. The exceptions are PBE+D3, for which the volumes differ by 0.5%, and bare PBE, for which the difference is a much larger 6.2%. This can be attributed to the steeper curvature in the FFLUX calculations, making the location of the minimum more precise and, by the same token, the shallow curvature in the PBE calculation, making the location somewhat less precise.

**Table 5:** Equilibrium Total Energies *E*_0_, Volumes *V*_0_, Bulk Moduli *B*_0_, and Pressure Derivatives  Obtained by Fitting Energy–Volume Curves to the Birch–Murnaghan Equation of State (eq 15)^a^

method	*E*_0_/kJ mol^–1^ molecule^–1^	*V*_0_/Å^3^ molecule^–1^	*V*_opt_/Å^3^ molecule^–1^	*B*_0_/GPa	
FFLUX *L*′ = 0	–446,432.69	51.16	51.17	18.11	10.23
FFLUX *L*′ = 1	–446,336.47	57.85	57.83	8.12	9.23
FFLUX *L*′ = 2	–446,341.72	56.54	56.55	9.87	11.37
PBE	–3468.16	72.31	68.11	0.83	15.18
PBE+D3	–3496.02	55.91	55.64	4.99	2.92

a The volumes of the optimized cells, *V*_opt_, are also given for comparison to the fitted *V*_0_. The derivative of the bulk modulus with respect to the pressure, , is dimensionless.

A comparison between the curves obtained with PBE+D3 and PBE suggests that the dispersion correction in the former has a large impact on the curvature. Hence, we again attribute the difference between the PBE+D3 and FFLUX curves with converged electrostatics to the representation of the nonbonded interactions in FFLUX. Following on from our previous conclusion about the nonbonded parameters impacting the reproduction of the β formamide structure, it is likely that the changing environment within the crystals upon expanding and compressing the volume is not captured well by a single set of Lennard-Jones parameters.

### 4.3 Phonon Calculations

**Figure 4:**
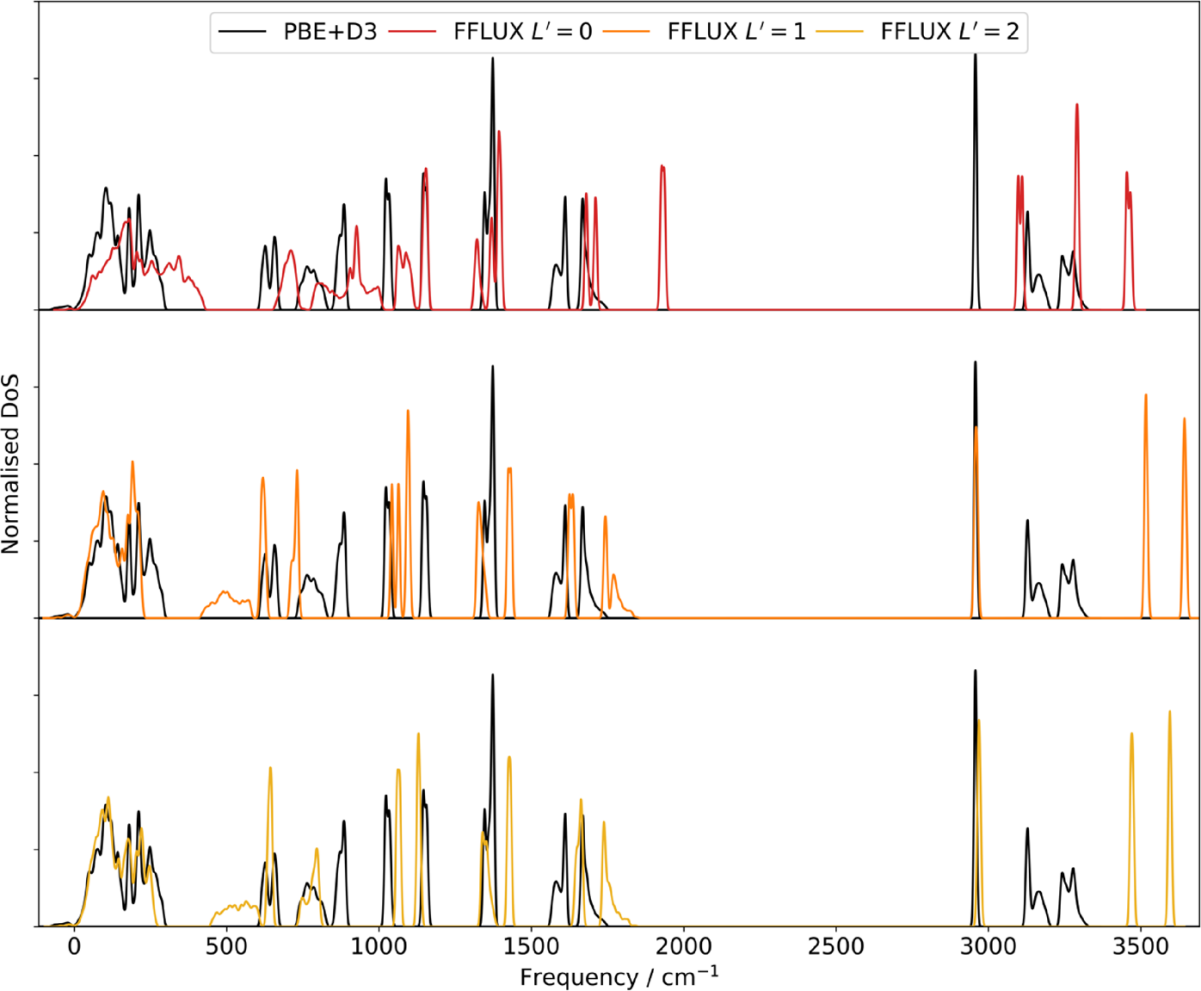
Comparison of the PBE+D3 phonon density of states (DoS) of α formamide to the FFLUX DoS obtained with *L*′ = 0 (top, red), *L*′ = 1 (middle, orange), and *L*′ = 2 (bottom, yellow).

Phonon DoS curves based on forces calculated using PBE+D3 and FFLUX were obtained for both formamide phases, with the FFLUX calculations coming at a significantly lower computational cost. Section 3 of the Supporting Information estimates the time VASP would require for the 2016-atom supercell used in FFLUX calculations and gives a comparison of the time taken for different multipolar ranks (Table S3.1). Figure 4 compares the PBE+D3 DoS of the α phase to FFLUX simulations performed with different multipolar ranks. The DoS for the β phase is given in Section 4 of the Supporting Information (Figure S4.1). Upon increasing *L*′, we see that the frequencies of the features in the DoS begin to converge toward values that are generally closer to the PBE+D3 result. This is perhaps to be expected, given that the better representation of the electrostatic interactions between molecules obtained using higher-order multipole moments should lead to more accurate force calculations. However, it should be noted that the aim of this exercise was primarily to establish whether FFLUX can produce sufficiently accurate and noise-free forces to be used in phonon calculations, rather than to accurately reproduce the PBE+D3 DoS. This is because the predominantly intramolecular modes at higher frequencies are largely described using the GPR model. The model is trained using a different level of theory to the periodic DFT calculations (B3LYP/aug-cc-pVTZ versus PBE+D3 with a plane wave basis), and therefore, differences between the phonon spectra calculated by the two methods should be expected.

As a reference point for interpreting the phonon spectra, we performed a frequency calculation on a gas-phase formamide monomer using B3LYP/aug-cc-pVTZ in GAUSSIAN09 and assigned the bands (Table 6). The calculated frequencies are in line with a previous study.^65^ As changes in vibrational frequencies are expected going from the gas phase to a crystal, PBE+D3 frequencies were also calculated using VASP and Phonopy with an 850 eV plane-wave cutoff and a 1 × 1 × 1 **k**-point mesh. The PBE+D3 frequencies are given in Section 5 of the Supporting Information (Table S5.1).

**Table 6:** Vibrational Frequencies of the Formamide Monomer Calculated at the B3LYP/aug-cc-pVTZ Level of Theory

frequency/cm^–1^	assignment
260	NH_2_ wag
569	NCO bend
639	NH_2_ torsional twist
1046	CH out-of-plane bend
1054	NH_2_ in plane bend
1265	CN stretch
1418	CH bend
1617	NH_2_ scissor
1786	C=O stretch
2941	CH stretch
3575	NH_2_ symmetric stretch
3709	NH_2_ asymmetric stretch

The position of the CH stretch significantly changes on increasing *L*′, moving from above 3000 cm^–1^ at *L*′ = 0 to 2969 cm^–1^ at *L*′ = 2. The latter frequency agrees well with both PBE+D3 (2957 cm^–1^) and the gas-phase monomer calculation (2941 cm^–1^). From this agreement, it can be inferred that, at the very least, dipole moments are required in simulations to accurately capture the impact of the crystal environment on this mode. Better representations of the electrostatics also provide a better representation of the low frequency modes, which is important for the calculation of the vibrational free energy.

In the *L*′ = 2 DoS, the symmetric and asymmetric NH_2_ stretches at 3470 and 3595 cm^–1^ are predicted at a higher frequency than with PBE+D3. The higher frequencies can be accounted for by the use of B3LYP/aug-cc-pVTZ to train the GPR model (cf. Table 6), for which the stretches occur at higher frequencies than when PBE+D3 is used (cf. Table S5.1). The gas phase stretch frequencies are approximately 200 cm^–1^ higher than those in the FFLUX solid-state calculations, which can be explained by the hydrogen bonding in the crystal. Hydrogen bonding is well known to cause a red shift of the in-plane NH vibrational frequencies.

The features between ∼600 and 900 cm^–1^ in the PBE+D3 DoS are due to, from low to high frequency, the NCO bend, NH_2_ wagging, and the NH_2_ torsional twist vibrations. In the FFLUX DoS, these peaks occur over a lower range of ∼450–800 cm^–1^ and the order of the NH_2_ wag and NCO bend vibrations is reversed. Given that both monomer calculations (B3LYP and PBE+D3) agree with the ordering seen in the FFLUX DoS, the rearrangement cannot be put down solely to the training level of theory. The rearrangement in the PBE+D3 DoS could instead be partly due to the crystal packing, causing some modes to be blue-shifted more than others. Each of the three modes is blue-shifted relative to the gas phase calculations, which is consistent with a report that hydrogen bonding can cause out-of-plane bends to be blue-shifted.^66^ GIF animations of the modes discussed here are provided in the Supporting Information.

We note that there are several alternatives to the finite-displacement method used in these phonon calculations, among which is the temperature-dependent effective potential (TDEP) method,^67^ which evaluates the force constants at finite temperature based on molecular-dynamics data. This is notable because FFLUX can be used to generate these trajectories at a significantly smaller computational cost than ab initio molecular dynamics. While we do not pursue this here, we intend to explore the possibility in future work.

Furthermore, the description of volume-dependent properties such as the Gibbs free energy requires the use of the quasi-harmonic approximation, which applies the harmonic approximation to a series of compressed and expanded crystal structures and accounts for the volume dependence of the phonon frequencies. In the work presented here, we consider only the harmonic approximation, but in principle, the FFLUX Phonopy workflow can be extended to the quasi-harmonic approximation. Indeed, the fact that the calculation of energy–volume curves is possible with FFLUX indicates that quasi-harmonic calculations should be easily accessible.

### 4.4 Infrared Spectra

**Figure 5:**
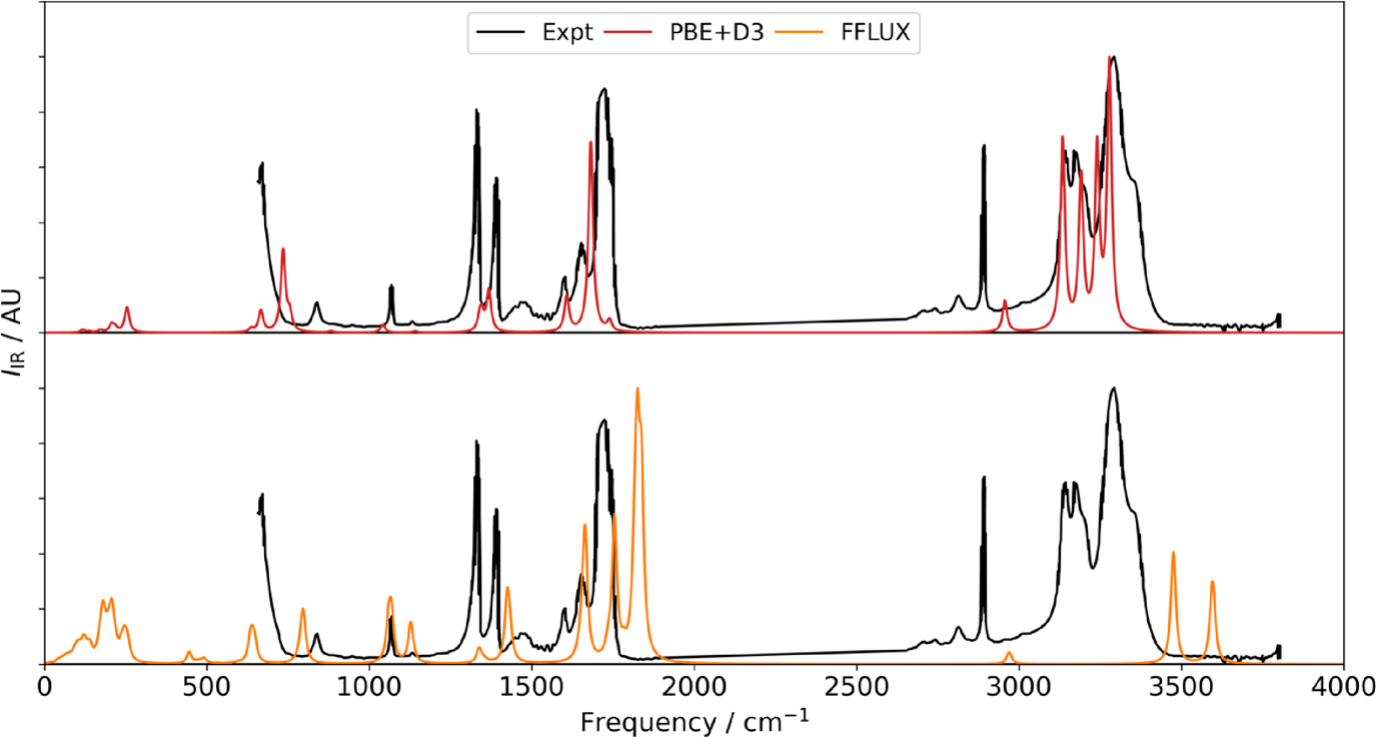
IR spectra of α formamide calculated using PBE+D3 (top, red) and FFLUX (bottom, orange) compared to experimental data from Sivaraman et al.^69^

Calculation of the phonon frequencies also allows the infrared (IR) spectra to be modeled and facilitates a direct comparison to experimental data. This formally requires the so-called “Born charges” (displacement dipoles) Z*, which are 3 × 3 tensors that describe the change in polarization (dipole moment per unit volume) when an atom is displaced along the three Cartesian directions. The PBE+D3 IR spectrum was calculated by combining the frequencies and eigenvectors from the dynamical matrix evaluated at **q** = Γ with the Phonopy package and Z* obtained from the DFPT routines in VASP.^68^ For the FFLUX calculations, the solid-state polarization **P** was obtained by calculating and summing the molecular dipole moment of each molecule in the supercell. The Z* were obtained by displacing each atom by ±0.01 Å along each Cartesian direction. The corresponding changes in polarization were then calculated, and the derivatives were evaluated with a two-point finite difference stencil. This is similar to the molecular dynamics (MD)-based approach in the study by Symons and Popelier.^23^ We note that this is only possible because of the geometry-responsive charges and dipole moments available from the GPR models in FFLUX. The IR spectra obtained using PBE+D3 and FFLUX are compared to experimental data taken from Sivaraman et al.^69^ in Figure 5.

As with the phonon density of states, differences in frequencies can be put down to the level of theory used, with the B3LYP-based GPR model producing higher frequencies than the experiments, while PBE+D3 captures the experimental peak positions well. The low-frequency modes are captured reasonably well with FFLUX, but at higher frequencies, the relative intensities of the CH and NH stretches are lower than in the experimental data and the PBE+D3 spectrum. The gas-phase monomer spectra generated using PBE+D3, B3LYP/aug-cc-pVTZ, and FFLUX indicate that PBE+D3 and B3LYP/aug-cc-PVTZ predict very similar relative peak intensities, whereas the intensities calculated using FFLUX with the Z* obtained from the finite-difference method are notably different (Figure S6.1 in Section 6 of the Supporting Information).

There are two possible origins for these discrepancies: that the eigenvectors predicted by FFLUX are different to those predicted by the two DFT methods and/or that the calculated Z* differ. To test for the former, the eigenvectors of each of the 12 vibrational modes of the monomers were compared using the cosine distance, *d*, given by
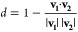
16Here, **v_1_** and **v_2_** are the 3*N* component vectors obtained by “flattening” the 3 × *N* eigenvectors. The cosine distance varies between 0 and 2, indicating that the two vectors are parallel and antiparallel, respectively. By this metric, the eigenvectors of the FFLUX monomer calculation were very similar to those obtained from both the PBE+D3 and B3LYP (which were also similar to each other). The similarity suggests that the differences in the IR intensities are due to either the way the Born charges have been calculated or to the predicted charges and dipole moments used to compute the polarization. Given the previously demonstrated accuracy of the formamide model,^24^ we suggest that the former is more likely, and we note that the alternative MD-based approach used in our previous study may give better results than the finite-difference method employed here.^23^

Whichever method is chosen, the ability to straightforwardly predict the IR spectra is another advantage of FFLUX, particularly in the context of polymorphism, where the simulated spectra could be used to experimentally verify predictions. While computationally more demanding than the finite-difference approach, MD simulations of appropriate length are accessible with FFLUX and would also have the advantage of capturing frequency shifts and line widths due to anharmonicity at finite temperature.

### 4.5 Thermodynamic Stability

**Figure 6:**
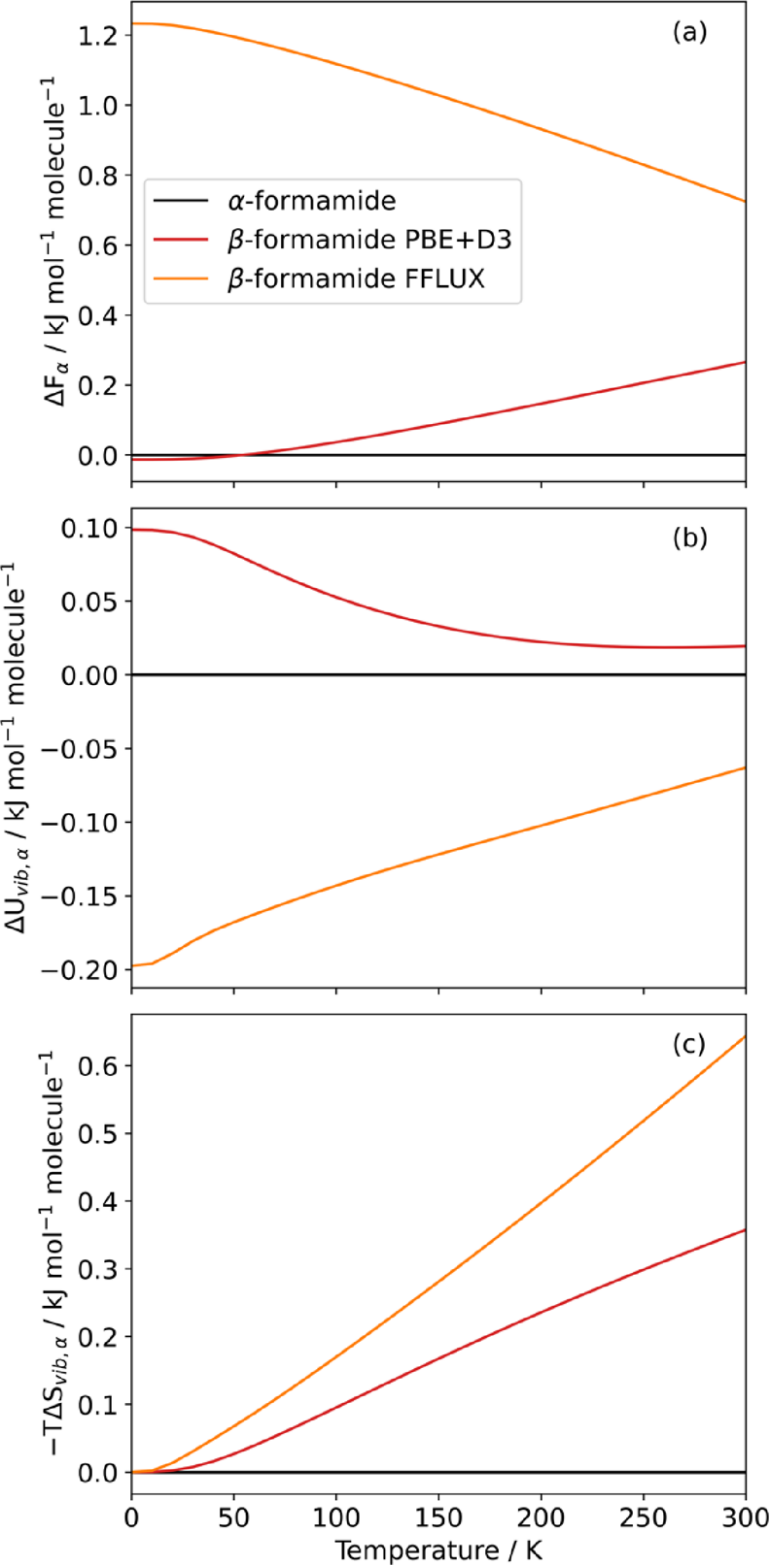
(a) Helmholtz energy of β formamide relative to the α phase ( Δ*F*_α_) calculated using PBE+D3 and FFLUX (*L*′ = 2). (b, c) Breakdown of the PBE+D3 and FFLUX energy differences into contributions from the vibrational free energy (b) and vibrational entropy (c).

In a CSP study, it is common to rank potential crystal structures by their lattice energy, with the lowest energy structures assumed to be the ones accessible in experimental syntheses. With the phonon calculations performed here, it is also possible to access the temperature-dependent Helmholtz free energy *F*. Figure 6a compares the calculated Helmholtz energy difference between the ambient α and β formamide structures (β – α), obtained using PBE+D3 and FFLUX, with Δ*F*_α_ representing the Helmholtz free energy relative to the α phase.

FFLUX predicts the α phase to be the most stable across the temperature range examined, which agrees with the chemical intuition that the reported ambient-pressure structure should be more stable than a high-pressure structure at ambient pressure. Interestingly, the PBE+D3 calculations contradict intuition by predicting that the high-pressure β phase to be more stable at temperatures below 50 K, although the actual free energy difference is an order of magnitude smaller than chemical accuracy (<0.1 kJ mol^–1^ versus 4.18 kJ mol^–1^). The separate contributions to the Helmholtz energy from the vibrational internal energy and entropy (cf. eq 9) reveal that both phonon terms disfavor β formamide at low temperature, indicating that the low-temperature stability of the β phase predicted by PBE+D3 is due to a lower lattice energy. On the other hand, the FFLUX calculations predict the high-pressure β phase to be less stable than the α phase at all temperatures. The FFLUX simulations predict a similar behavior to PBE+D3 in the vibrational entropy of the β phase, with the entropy being destabilizing with increasing temperature. However, a significant qualitative difference is seen in the vibrational internal energy, with FFLUX predicting this contribution to be stabilizing, albeit increasingly less so with temperature. Cumulative DoS curves (Figure S7.1 in Section 7 of the Supporting Information) show that for the PBE+D3 calculations, the high frequency modes in the α phase occur at slightly lower frequencies than in the β phase. However, the cumulative free energy as a function of frequency (Figure S7.2) suggests that the differences in the free energy cannot be attributed to any one region of the DoS.

## 5 Conclusions

Numerous methods to study molecular crystals have been developed over the past few decades, but among a diverse set of force field methods, a common approximation is the use of point charges or multipole moments but with rigid-body representations of molecules. By using Gaussian process regression (GPR) to predict atomic energies and atomic multipole moments based on local geometry, the FFLUX force field overcomes the rigid-body limitation imposed on the current methods used in crystal structure prediction. This work shows that the combination of the GPR models used in FFLUX simulations with a parametrized dispersion correction can produce accurate results in solid-state calculations, allowing for good quality geometry optimizations and also lattice dynamics and free energy calculations within the harmonic approximation.

Of particular note are the optimizations of α formamide with FFLUX that highlighted the importance of including higher-order multipole moments in simulations. When only charges were used, the lattice parameters differed significantly from the experimental structure, whereas the inclusion of both dipole and quadrupole moments led to differences of less than 3%, which were comparable to PBE+D3 calculations. The multipolar rank also had a significant effect on the RMSE compared to the experimental structures but did not correct previously identified problems with the inter-ring angles in the crystal structure, which we attribute to deficiencies in the nonbonded parameters. Optimization of the high-pressure β phase was not as successful but was still possible within the FFLUX methodology.

Calculation of energy–volume curves was found to be possible with FFLUX, albeit with a steeper curvature than in DFT methods, leading to an overprediction of the bulk moduli. This is again attributed to the nonbonded parameters, which we believe are unable to adapt to a changing environment in the way that the *C*_6_ parameters in the Grimme D3 dispersion correction can.

The importance of higher-order multipole moments is also shown in the lattice dynamics calculations. In particular, higher order multipole moments allowed the low frequency intramolecular modes to be captured well, compared to PBE+D3. Other significant differences in the higher-frequency density of states, corresponding to predominantly intramolecular modes, could be accounted for by the different training level of theory used to parametrize the GPR models. Taken together with the reasonable reproduction of the equation-of-state curves, the present work indicates that it could be possible to obtain reasonable free energies with other techniques such as the quasi-harmonic approximation and molecular dynamics-based methods such as TDEP. The access to molecular dipole moments from atomic charges and dipole moments also allows for prediction of infrared intensities and the calculation of the infrared spectra.

Overall, this work shows that FFLUX models parametrized from single molecules are capable of performing reasonably accurate calculations on solids “out of the box” and can be used to model a range of solid-state properties. In particular, good quality energetics and phonon calculations, which provide access to free energies, should make this approach well suited to crystal-structure prediction, where we would expect FFLUX calculations to show superior performance to traditional force fields at a significantly smaller cost compared to DFT methods. The main issues in the FFLUX calculations appear to be attributable to the representation of dispersion and repulsion with a relatively simple nonbonded potential.

Within the FFLUX framework, it is in principle possible to eliminate the need for such potentials and incorporate nonbonded interactions into the machine learning models. This is a nontrivial machine learning problem but, if solved, would allow for more accurate calculations, bringing FFLUX even closer to quantum mechanics.

## Data Availability

The data supporting the findings reported in this paper are openly available from the “Data for: *Application of the FFLUX Force Field to Molecular Crystals: A Study of Formamide*” repository at doi: 10.17632/whw57k6d33.1.

## Supporting Information Available

Determination of the nonbonded Lennard-Jones parameters (Tables S1.1 and S1.2); RMSE of optimized α and β formamide compared to the experimental structures (Table S2.1); time taken for FFLUX force calculations at different *L*′ (Table S3.1); phonon density of states of β formamide calculated using FFLUX (*L*′ = 2) and PBE+D3 (Figure S4.1); vibrational frequencies of the formamide monomer calculated using PBE+D3 (Table S5.1); IR spectra of a gas phase formamide monomer calculated using PBE+D3, B3LYP/aug-cc-pVTZ, and FFLUX compared to experiment (Figure S6.1); cumulative density of states curves for the two phases of formamide calculated using PBE+D3 (Figure S7.1); cumulative free energy as a function of frequency (Figure S7.2) (PDF)
GIF animations of selected phonon modes discussed in the text (ZIP)
