# Supplementary material for: Application of the FFLUX Force Field to Molecular Crystals: A Study of Formamide
Source: J Chem Theory Comput. 2023 Oct 17;19(21):7946–59. doi: 10.1021/acs.jctc.3c00578 (PMC10653110; doi:10.1021/acs.jctc.3c00578)
Supplement: Supplementary file 1 — ct3c00578_si_001.pdf [file ct3c00578_si_001.pdf]

Supporting Information

Application of the FFLUX Force Field to Molecular Crystals: A Study of Formamide

Matthew L. Brown, Jonathan M. Skelton and Paul L. A. Popelier*

Department of Chemistry, The University of Manchester, Oxford Road, Manchester, M13 9PL, United Kingdom

*Phone: +44 161 3064511. Email: pla@manchester.ac.uk

Contents

**1**	**Optimisation of Non-Bonded Parameters**	S2
**2**	**RMSE of Optimised Crystal Structures**	S5
**3**	**Evaluation of Computational Cost**	S6
**4**	**Density of States of High-Pressure Phase**	S8
**5**	**PBE+D3 Monomer Vibrational Frequencies**	S9
**6**	**Gas Phase Monomer IR Spectra**	S10
**7**	**Cumulative Density of States**	S11
	**References**	S13

1 Optimisation of Non-Bonded Parameters

When monomeric Gaussian process regression (GPR) models are used in FFLUX simulations, a non-bonded potential is required to model non-electrostatic intermolecular interactions. Several different potentials are available in DL_POLY and hence DL_FFLUX. In this work a 12-6 Lennard-Jones potential was used:

$$U(r_{ij}) = \frac{A_{ij}}{r_{ij}^{12}} - \frac{B_{ij}}{r_{ij}^{6}} \tag{S1.1}$$

where A_{ij} and B_{ij} are parameters for the interactions between atoms i and j. We used as our initial non-bonded parameters those derived by Hagler *et al.* [1], which are given in Table S1.1.

Table S1.1. Initial non-bonded parameters derived by Hagler *et al.*

Atom	A / kJ mol^{-1} Å^{12}	B / kJ mol^{-1} Å^{6}
C	12,644,048.000	5606.560
N	9,501,864.000	5146.320
O	1,150,600.000	2100.368

As described in reference [1] the following mixing rules were used to obtain parameters for the interactions between pairs of atoms of different types:

$$A_{ij} = \sqrt{A_{ii}A_{jj}} \tag{S1.2}$$

$$B_{ij} = \sqrt{B_{ii}B_{jj}} \tag{S1.3}$$

Following the protocol presented in reference [2], these initial non-bonded parameters were optimised for use in $L' = 2$ simulations. In these simulations, monopole, dipole and quadrupole moments are used to describe the intermolecular electrostatic interactions. The parameters were adapted by scaling both the A and B values by a factor n such that scaled parameters A_{ij}^{*} and B_{ij}^{*} are given by:

$$A_{ij}^{*} = nA_{ij} \tag{S1.4}$$

$$B_{ij}^{*} = nB_{ij} \tag{S1.5}$$

Optimisations using each of the non-bonded parameter sets were performed as described in the main text. The density (ρ), lattice energy (U_{Latt}) and β angle of each optimised structure was then calculated and scored against the experimental values of 1.298 g cm^{-3}, -79.2 kJ mol^{-1} [3] and 98°, respectively. The scoring was done using the weighted square difference:

$$S = \sum_{p} w_p \left(\mathrm{Calc}_p - \mathrm{Exp}_p\right)^2 \tag{S1.6}$$

with $w_\rho = 3$, $w_{U_{Latt}} = 1$ and $w_\beta = 5$. The lowest weight was assigned to the lattice energy due to the magnitude of the difference being the largest, while the largest weight was assigned to the β angle. This is despite the magnitudes of the differences in the angles being larger than in the densities, hence putting a greater importance on getting the angle closer to experiment.

The best performing parameter set is the one that minimises the score, with a score of 0 indicating that all experimental values have been reproduced exactly. The values obtained for each of the properties with each parameter set are given in Table S1.2. The best performing set was found to be the Hagler parameters increased by 40%, which are highlighted in bold in the table.

Table S1.2. Dependence of ρ (g/cm^3), U_{Latt} (kJ mol^{-1}), and β (degrees) on the scale factor n applied to the initial set of non-bonded parameters derived by Hagler *et al.*. We highlighted in bold the chosen parameter set with the smallest score (S), which best reproduces the experimental parameters (shown in the utmost right column of the table).

Scaling n	ρ	U_{Latt}	β	S
0.900	1.366	-67.753	95.751	156.343
0.925	1.362	-68.136	95.930	143.854
0.950	1.359	-68.529	96.121	131.530
0.975	1.356	-68.932	96.305	119.802
1.000	1.354	-69.344	96.473	108.797
1.025	1.351	-69.765	96.663	97.971
1.050	1.348	-70.193	96.837	87.887
1.075	1.346	-70.629	97.000	78.469
1.100	1.344	-71.072	97.172	69.493
1.125	1.341	-71.522	97.335	61.169
1.150	1.339	-71.978	97.504	53.401
1.175	1.337	-72.439	97.679	46.229
1.200	1.335	-72.906	97.843	39.736
1.225	1.333	-73.379	98.022	33.891
1.250	1.332	-73.856	98.183	28.723
1.275	1.330	-74.339	98.366	24.307
1.300	1.328	-74.826	98.525	20.518
1.325	1.327	-75.317	98.679	17.385
1.350	1.325	-75.812	98.846	15.061
1.375	1.324	-76.312	99.019	13.532
**1.400**	**1.322**	**-76.815**	**99.191**	**12.786**
1.425	1.321	-77.322	99.368	12.888
1.450	1.320	-77.832	99.533	13.627
1.475	1.319	-78.345	99.708	15.314
1.500	1.317	-78.863	99.867	17.548
1.525	1.316	-79.382	100.047	20.986
1.550	1.315	-79.905	100.212	24.957
1.575	1.314	-80.431	100.385	29.955
1.600	1.313	-80.960	100.547	35.543
Exp	1.298	-79.200	98.000	

2 RMSE of Optimised Crystal Structures

Root-mean-square errors (RMSEs) between the experimental structures and the FFLUX optimised structures at different electrostatic ranks were calculated using the Kabsch algorithm to find the optimal translation and rotation to map the structures onto each other. These are given in Table S2.1.

Table S2.1. RMSE values of the optimised cells excluding hydrogen atoms compared to experiment. The label "hp" denotes a high-pressure optimisation performed at 1.2 GPa.

Method	RMSE (no H) / ˚A
α-formamide	
FFLUX $L' = 0$	2.11
FFLUX $L' = 1$	0.86
FFLUX $L' = 2$	0.38
PBE	0.18
PBE+D3	0.08
β-formamide	
FFLUX $L' = 2$	0.66
hp FFLUX $L' = 2$	0.83
PBE	0.23
hp PBE	1.01
PBE+D3	0.17
hp PBE+D3	0.24

3 Evaluation of Computational Cost

The phonon calculations performed in the main text were carried out by using the finite displacement method. In this method a series of supercells are generated in which each of the independent atoms are displaced from their ideal positions in the "perfect" supercell. A single-point calculation is then performed on each structure to evaluate the resulting atomic forces, which are then combined to derive the second-order interatomic force constants and to compute the phonon density of states and related thermal properties. Each of the FFLUX-optimised α formamide structures at $L' = 0$-2 required 12,096 force calculations. Table S3.1 shows the average time taken (core hr) for all 12,096 displacements and the relative cost compared to $L' = 0$.

Table S3.1. The average time taken for force calculations with FFLUX at different multipolar ranks. The time relative to $L' = 0$ (only charges in the electrostatic representation) is given.

L'	Average Time / core hr	Relative Cost
0	0.0153	1
1	0.0167	1.092
2	0.0278	1.817

The additional computational effort for including higher order multipole moments up to the quadrupole can be considered insignificant given that each calculation took of the order of seconds to complete.

The VASP calculations performed in the main text were carried out using a significantly smaller supercell than in the FFLUX calculations (384 atoms in VASP calculations *versus* 2016 atoms in FFLUX calculations). The time that VASP would require for the 2016 atom supercell can be estimated based on the time required for the 384 atoms supercell and the fact that VASP scales somewhere between N^2 and N^3 with the number of valence electrons (effectively, the number of atoms).

A VASP calculation using **128 cores** for **384 atoms** took **3456.95 s** while a FFLUX ($L' = 2$) calculation using **8 cores** for **2016 atoms** took **12.593 s**. From these numbers one can estimate how many times FFLUX is faster than VASP, as shown here:

Scaling of system size:

$$\frac{2016\ \text{atoms}}{384\ \text{atoms}} = 5.25$$

Converting to core hrs:

$$\text{VASP Time (hr)} = \frac{3456.95\ \text{s}}{3600\ \text{s hr}^{-1}} = 0.9603\ \text{hr}$$

$$\text{VASP Time (core hr)} = 128\ \text{core} \times 0.9603\ \text{hr} = 122.9\ \text{core hr}$$

$$\text{FFLUX Time (hr)} = \frac{12.593\ \text{s}}{3600\ \text{s hr}^{-1}} = 0.00350\ \text{hr}$$

$$\text{FFLUX Time (core hr)} = 8\ \text{core} \times 0.003450\ \text{hr} = 0.02800\ \text{core hr}$$

Estimate of the time taken for 2016-atom supercell with VASP (assuming N^2 scaling):

$$122.9\ \text{core hr} \times 5.25^2 = 3387.8\ \text{core hr}$$

$$\frac{3387.8\ \text{core hr}}{0.02780\ \text{core hr}} = 121{,}061\ \text{times slower than FFLUX}$$

Estimate of the time taken for 2016-atom supercell with VASP (assuming N^3 scaling):

$$122.9\ \text{core hr} \times 5.25^3 = 17{,}786\ \text{core hr}$$

$$\frac{17{,}786\ \text{core hr}}{0.0280\ \text{core hr}} = 635{,}568\ \text{times slower than FFLUX}$$

The 384-atom supercell in VASP required 122.9 core hr. The 2016-atom supercell is 5.2 times larger, which means that VASP would need between 3,388 core hr (assuming N^2 scaling) and 17,786 core hr (assuming N^3 scaling). We therefore estimate that FFLUX is between ~121,000 and ~635,000 times faster than VASP.

4 Density of States of High-Pressure Phase

The phonon density of states (DoS) of β-formamide (Figure S4.1) was calculated using the finite displacement method *via* the Phonopy package [4] with forces calculated using PBE+D3 and FFLUX with $L' = 2$. For the FFLUX calculations the atomic displacements and forces were evaluated in the simulation unit cell, which corresponds to a 7×2×4 expansion of the β-formamide unit cell (7x2x4x8x6=2688 atoms), and the phonon frequencies were evaluated on a 2 × 2 × 2 $\boldsymbol{q}$-point mesh. For the PBE+D3 calculations, the displacements and forces were evaluated in a 4×1×2 supercell of the unit cell (384 atoms), and frequencies were evaluated on a 16 × 16 × 16 $\boldsymbol{q}$-point mesh.

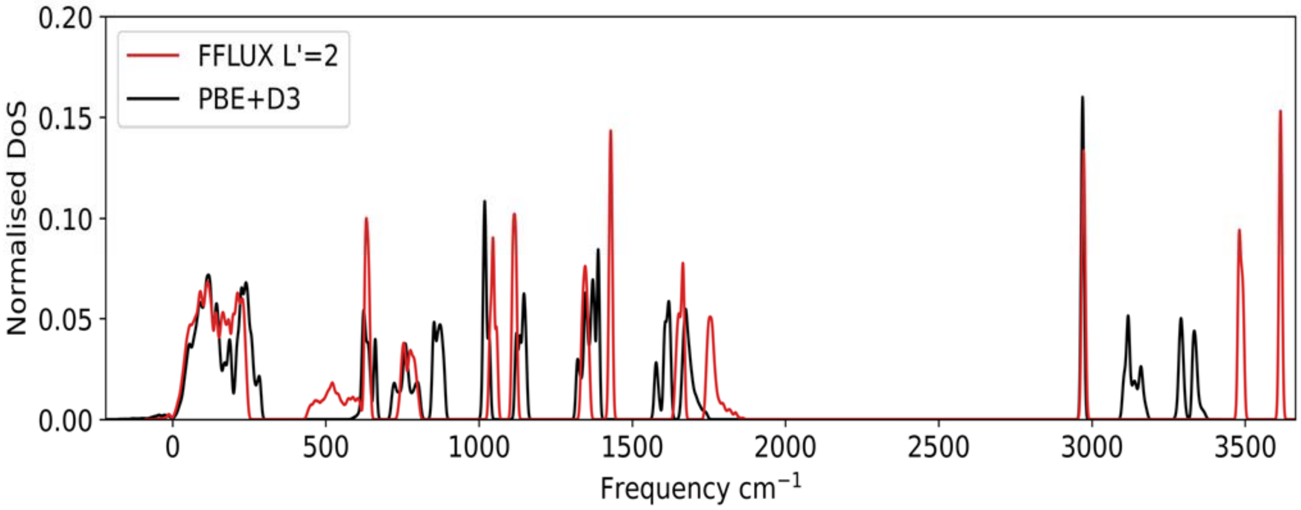

Figure S4.1. Phonon DoS of β-formamide obtained using PBE+D3 (black) and FFLUX at $L' = 2$ (red).

As noted in the main text, the purpose of these calculations was to determine whether it were possible to perform lattice dynamics calculations with FFLUX, and not necessarily to reproduce the PBE+D3 DoS. As discussed for the α phase in the main text, there are significant differences in the positions of some of the features, in particular those corresponding to predominantly intramolecular modes, which is due to the differences in levels of theory used in the periodic DFT calculations (i.e. PBE+D3) and in training the FFLUX GPR model (B3LYP/aug-cc-pVTZ). These differences are consistent with the ordering of the vibrational frequencies of a formamide monomer obtained at the training level of theory, presented in the main text, taking into account red- and blue-shifts in some modes due to the intermolecular interactions in the crystal.

5 PBE+D3 Monomer Vibrational Frequencies

Table S5.1. Vibrational frequencies of the formamide monomer calculated using plane wave PBE+D3.

Frequency / cm^{-1}	Assignment
264	NH_2 wag
546	NCO bend
632	NH_2 torsional twist
988	CH out of plane bend
1019	NH_2 in plane bend
1237	CN stretch
1360	CH bend
1557	NH_2 scissor
1741	C=O stretch
2854	CH stretch
3492	NH_2 symmetric stretch
3633	NH_2 asymmetric stretch

6 Gas Phase Monomer IR Spectra

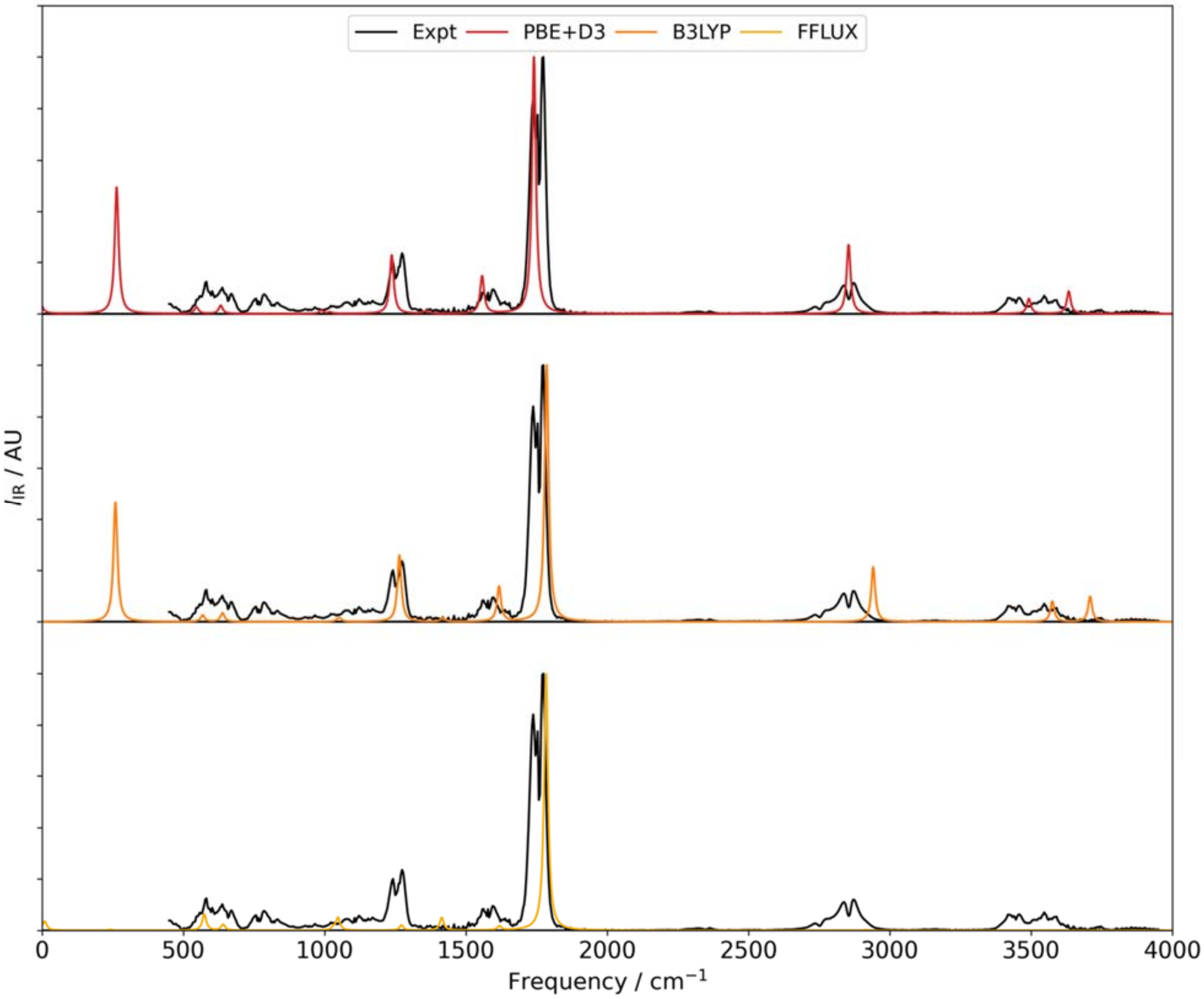

Figure S6.1. IR spectra of a gas phase formamide monomer calculated using PBE+D3 (top, red), B3LYP/aug-cc-pVTZ (middle, orange) and FFLUX (bottom, yellow) compared to experimental data from the NIST database [5].

7 Cumulative Density of States

In the main text we showed that PBE+D3 predicts the β phase to be more stable than the α phase at low temperature. Breaking down the Helmholtz free energy into its constituent components (see eq 9 of the main text) of vibrational internal energy and entropy showed that both energy and entropy favoured the α phase, even over the range of temperatures where the β phase was predicted to be more stable. This finding indicates that the only remaining term in eq 9, that is, the lattice energy φ must be driving the difference in stability. The destabilising effect from the phonon contributions to the free energy can be understood from the cumulative density of states shown in Figure S7.1.

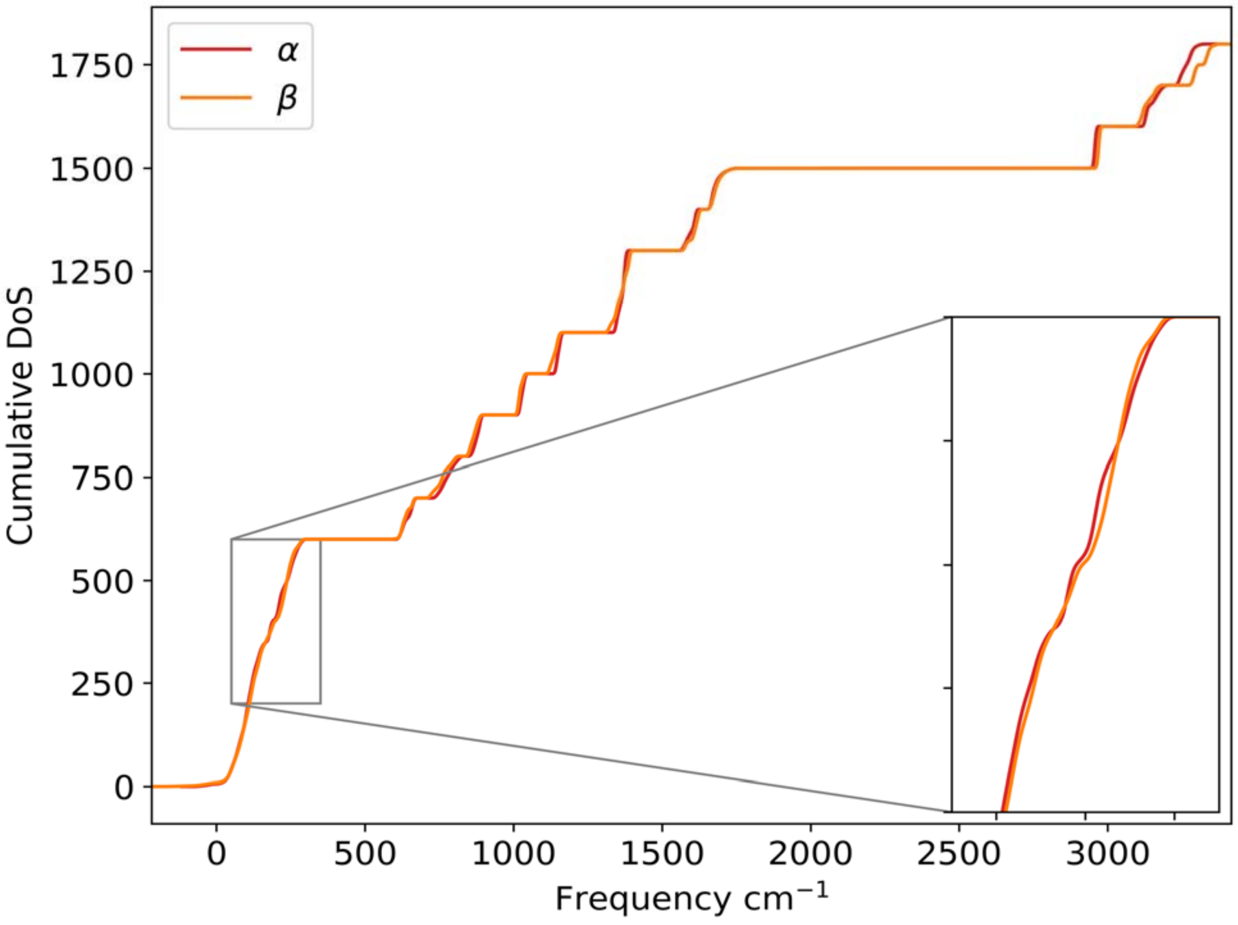

Figure S7.1. Cumulative DoS of α (red) and β formamide (orange) calculated using PBE+D3.

The high-frequency modes in the β phase occur at slightly higher frequencies than in the α phase, which increases the vibrational internal energy through the zero-point-energy (ZPE), calculated as

$$ZPE = \frac{1}{N}\sum_{\boldsymbol{q},j} \frac{\hbar}{2}\omega_{\boldsymbol{q},j} \tag{S7.1}$$

where $\omega_{\boldsymbol{q},j}$ is the frequency of a phonon with wavevector $\boldsymbol{q}$ and band index j while N is the number of $\boldsymbol{q}$ included in the Brillouin-zone sampling mesh. The difference in entropy between the two phases can be explained by the β phase having a lower density of modes at lower frequencies, which is highlighted in the zoomed-in section of Figure S7.1. The lower density of low-frequency modes means that β formamide has more high-lying modes than α formamide. By the partition function given in eq 8 of the main text, these higher-lying modes will contribute less to the partition function than their low frequency counterparts. In other words, the higher lying modes will have a lower occupation number, resulting in a lower vibrational entropy. However, the cumulative free energy as a function of the frequency shown in Figure S7.2 indicates that the differences in free energy between α and β formamide cannot be attributed to any one region of the DoS.

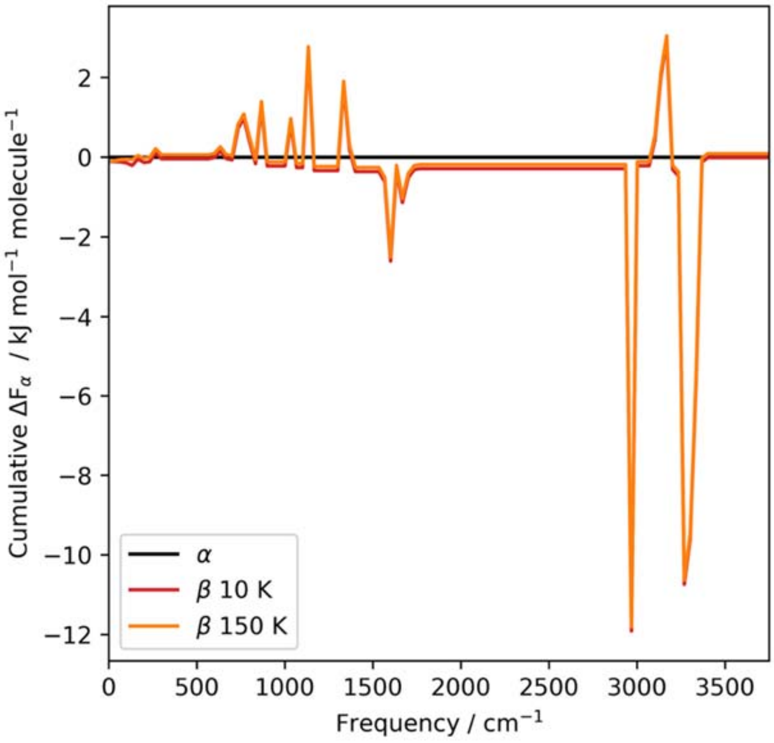

Figure S7.2. Cumulative free energy of β formamide at 10 (red) and 150 K (orange) relative to the α polymorph calculated using PBE+D3.

References

(1) Hagler, A. T.; Huler, E.; Lifson, S. *J.Am.Chem.Soc.* **1974**, *96*, 5319–5327.

(2) Hédin, F.; El Hage, K.; Meuwly, M. *J.Chem.Inf.Model.* **2016**, *56*, 1479–1489.

(3) Reilly, A. M.; Tkatchenko, A. *J.Chem.Phys.* **2013**, *139*, 024705.

(4) Togo, A.; Tanaka, I. *Scr. Mater.* **2015**, *108*, 1–5.

(5) *NIST Standard Reference Database Number 69.* https://webbook.nist.gov/cgi/cbook.cgi?ID=C75127 (accessed 2023-08-24)
