# Supplementary figures and images for: Application of the FFLUX Force Field to Molecular Crystals: A Study of Formamide
Source: J Chem Theory Comput. 2023 Oct 17;19(21):7946–59. doi: 10.1021/acs.jctc.3c00578 (PMC10653110; doi:10.1021/acs.jctc.3c00578)

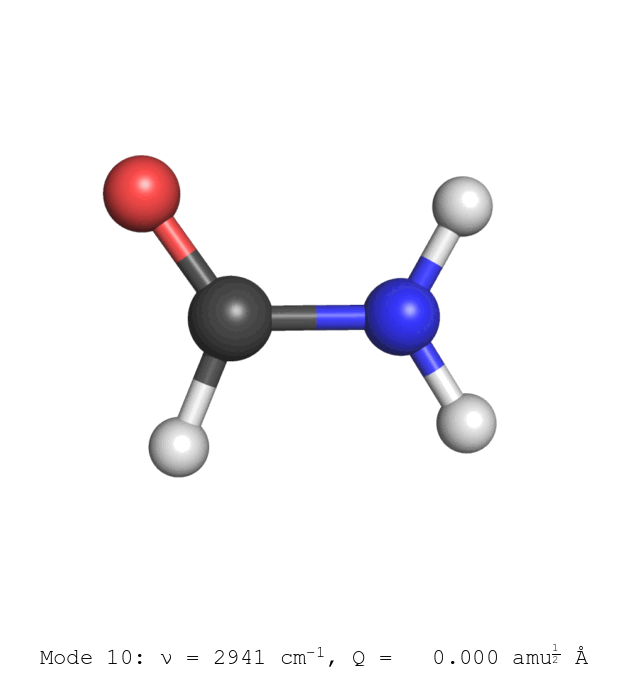

Supplement: Supplementary file 2 — ct3c00578_si_002.zip [file ct3c00578_si_002.zip › crystal_paper_gifs/B3LYP_MON_CH.gif]

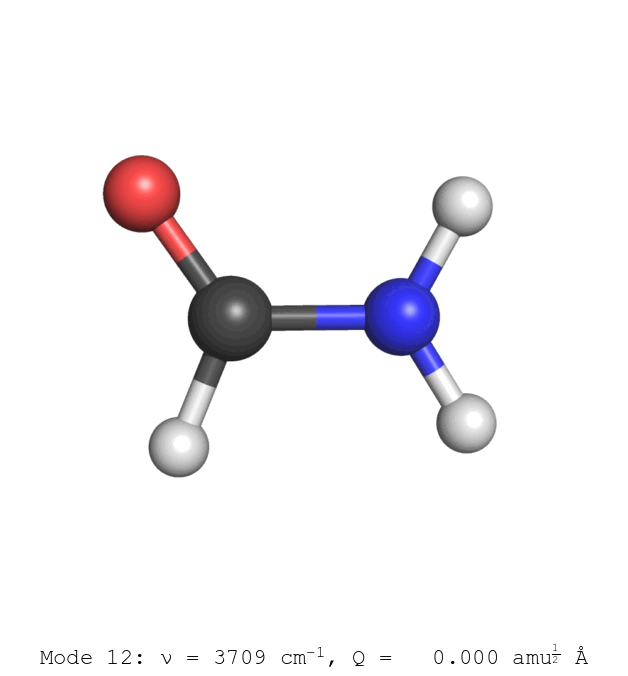

Supplement: Supplementary file 2 — ct3c00578_si_002.zip [file ct3c00578_si_002.zip › crystal_paper_gifs/B3LYP_MON_NHASYMM.gif]

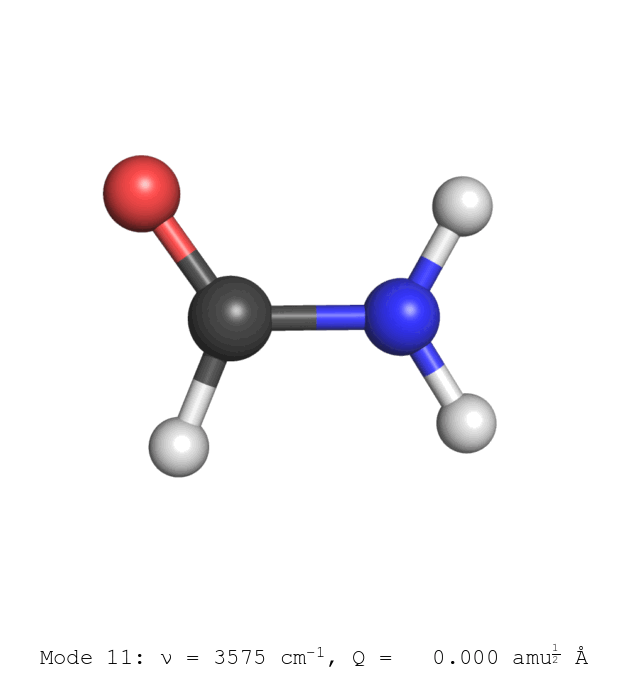

Supplement: Supplementary file 2 — ct3c00578_si_002.zip [file ct3c00578_si_002.zip › crystal_paper_gifs/B3LYP_MON_NHSYMM.gif]

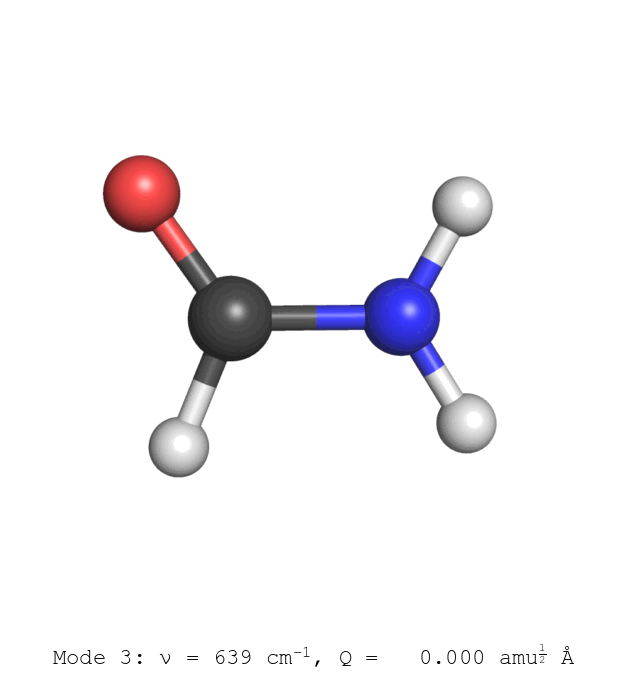

Supplement: Supplementary file 2 — ct3c00578_si_002.zip [file ct3c00578_si_002.zip › crystal_paper_gifs/B3LYP_MON_NHTWIST.gif]

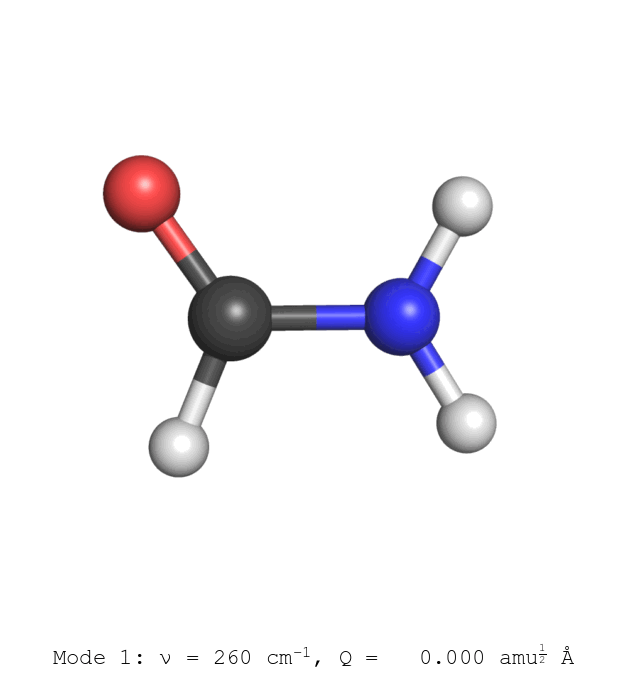

Supplement: Supplementary file 2 — ct3c00578_si_002.zip [file ct3c00578_si_002.zip › crystal_paper_gifs/B3LYP_MON_NHWAG.gif]

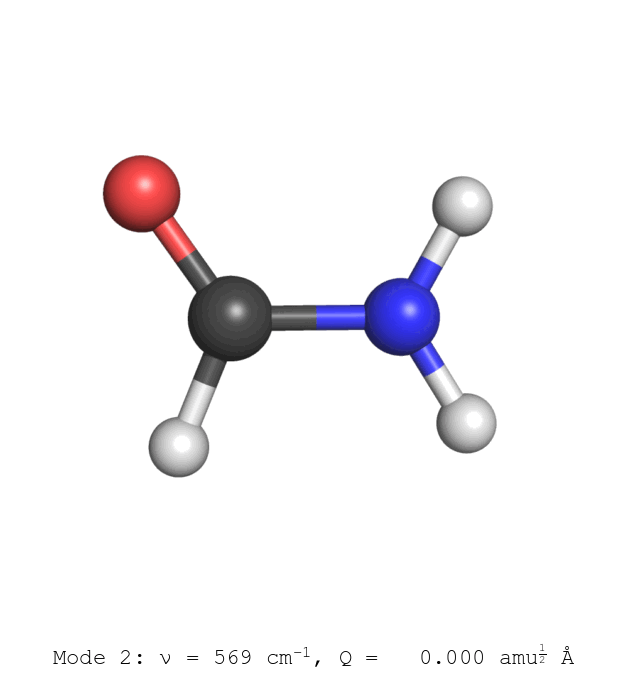

Supplement: Supplementary file 2 — ct3c00578_si_002.zip [file ct3c00578_si_002.zip › crystal_paper_gifs/B3LYP_MON_OCNBEND.gif]

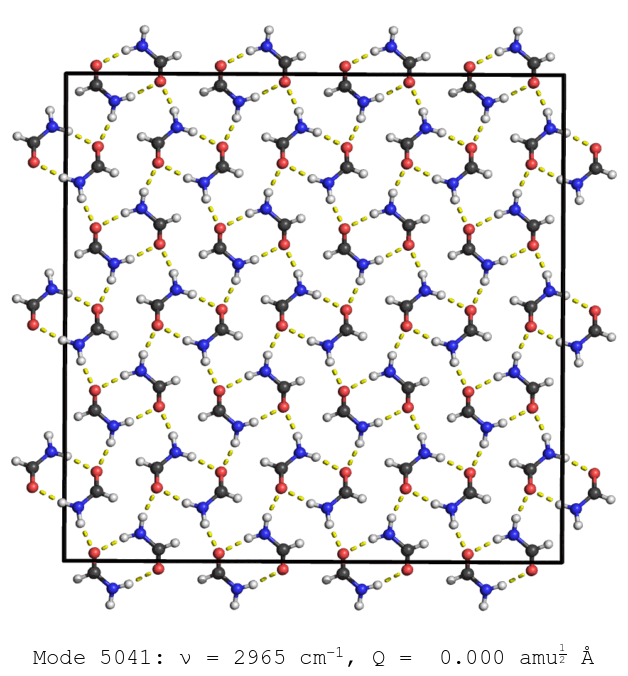

Supplement: Supplementary file 2 — ct3c00578_si_002.zip [file ct3c00578_si_002.zip › crystal_paper_gifs/FFLUX_A_CH.gif]

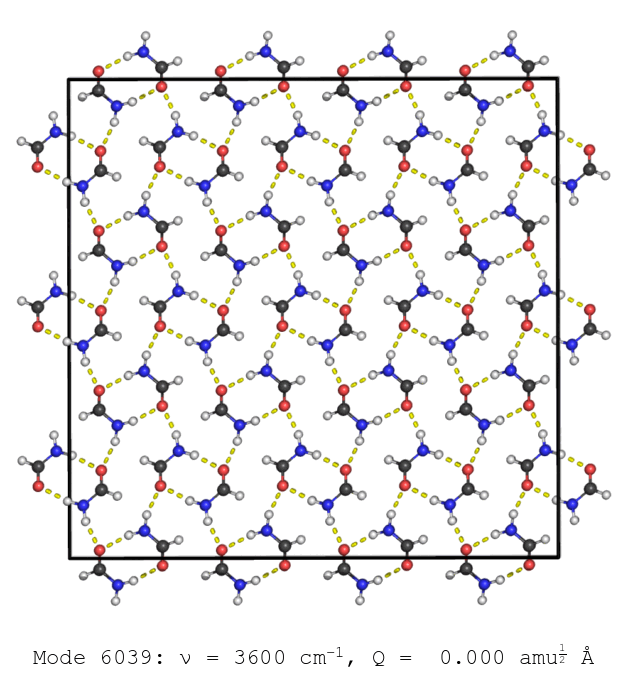

Supplement: Supplementary file 2 — ct3c00578_si_002.zip [file ct3c00578_si_002.zip › crystal_paper_gifs/FFLUX_A_NHASYMM.gif]

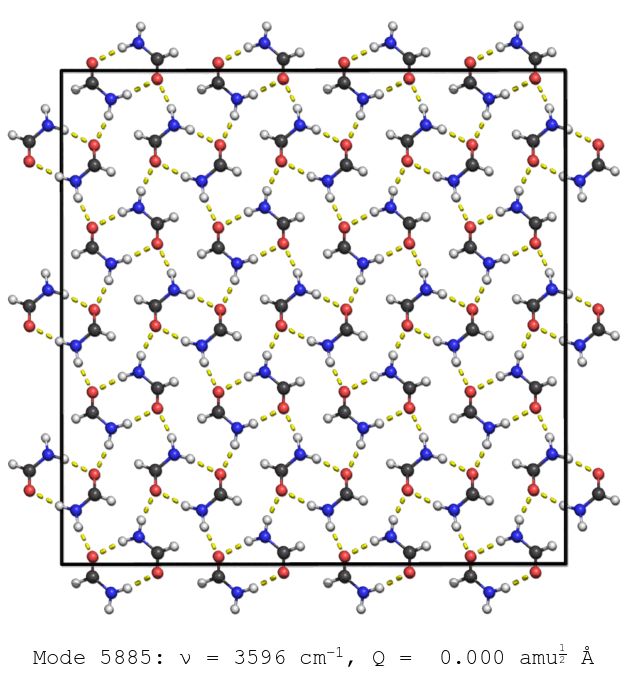

Supplement: Supplementary file 2 — ct3c00578_si_002.zip [file ct3c00578_si_002.zip › crystal_paper_gifs/FFLUX_A_NHSYMM.gif]

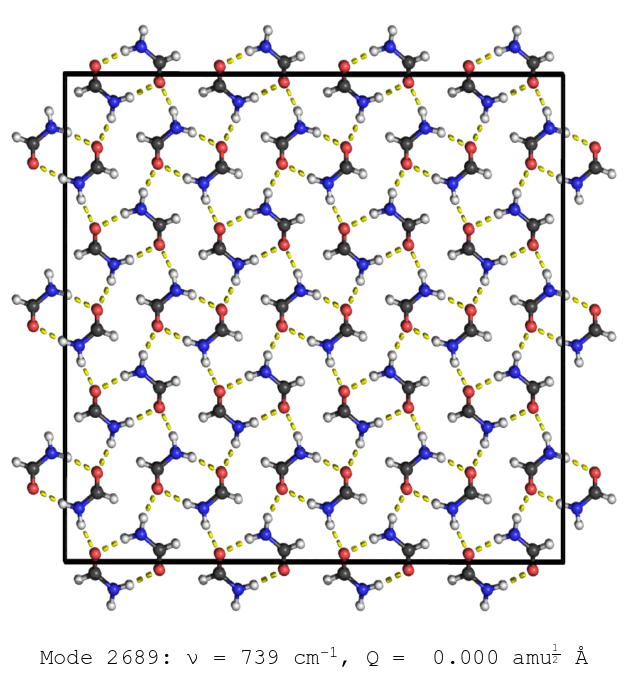

Supplement: Supplementary file 2 — ct3c00578_si_002.zip [file ct3c00578_si_002.zip › crystal_paper_gifs/FFLUX_A_NHTWIST.gif]

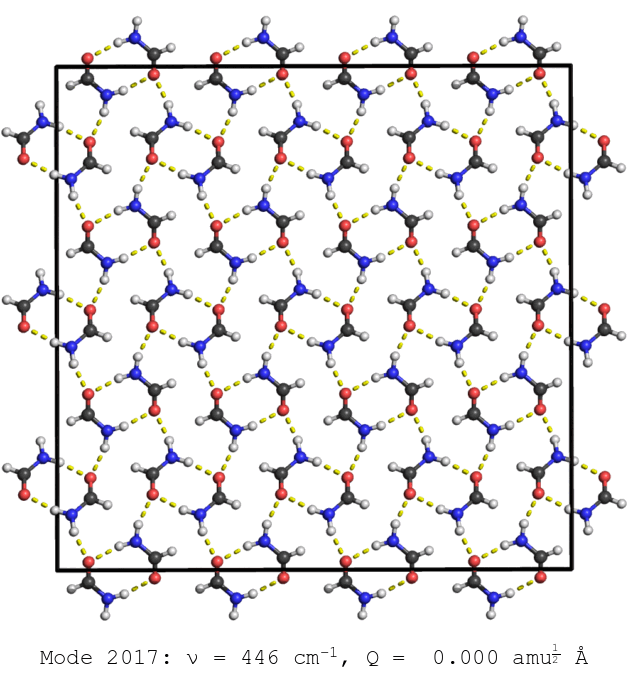

Supplement: Supplementary file 2 — ct3c00578_si_002.zip [file ct3c00578_si_002.zip › crystal_paper_gifs/FFLUX_A_NHWAG.gif]

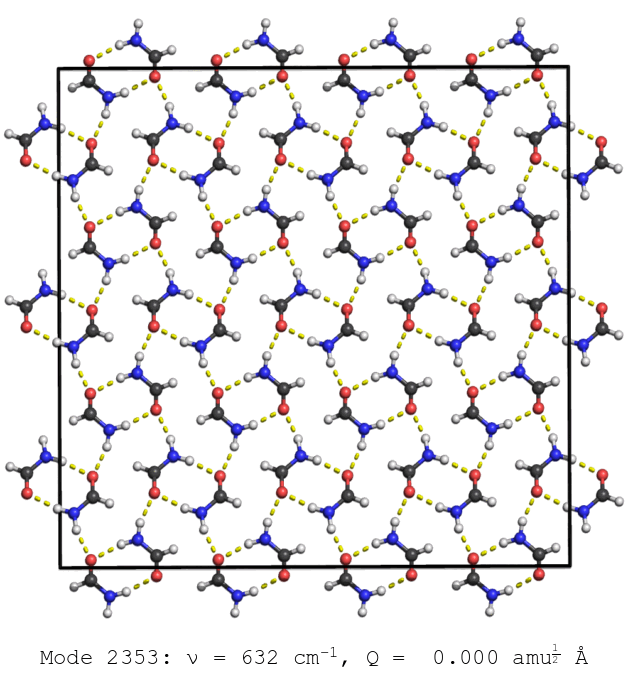

Supplement: Supplementary file 2 — ct3c00578_si_002.zip [file ct3c00578_si_002.zip › crystal_paper_gifs/FFLUX_A_OCNBEND.gif]

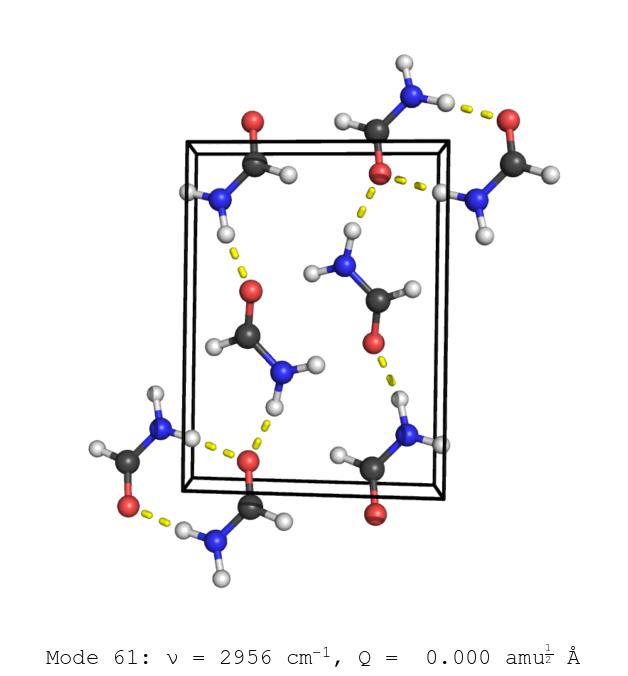

Supplement: Supplementary file 2 — ct3c00578_si_002.zip [file ct3c00578_si_002.zip › crystal_paper_gifs/PBED3_A_CH.gif]

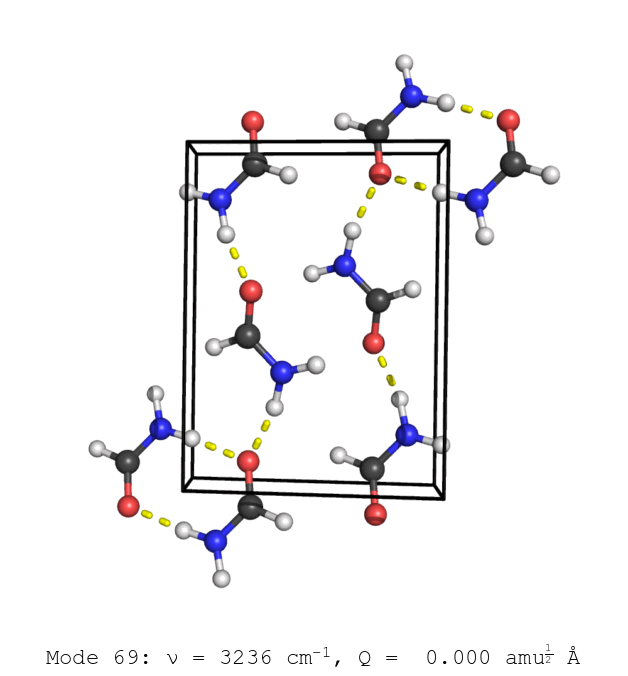

Supplement: Supplementary file 2 — ct3c00578_si_002.zip [file ct3c00578_si_002.zip › crystal_paper_gifs/PBED3_A_NHASYMM.gif]

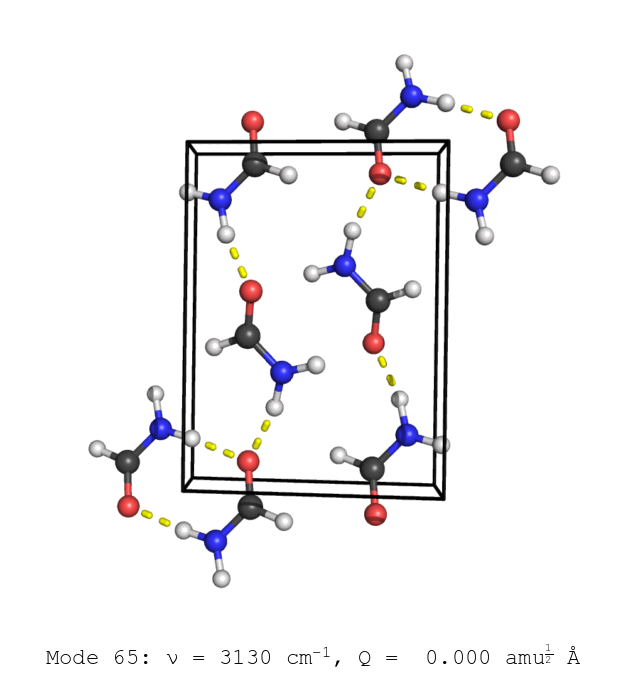

Supplement: Supplementary file 2 — ct3c00578_si_002.zip [file ct3c00578_si_002.zip › crystal_paper_gifs/PBED3_A_NHSYMM.gif]

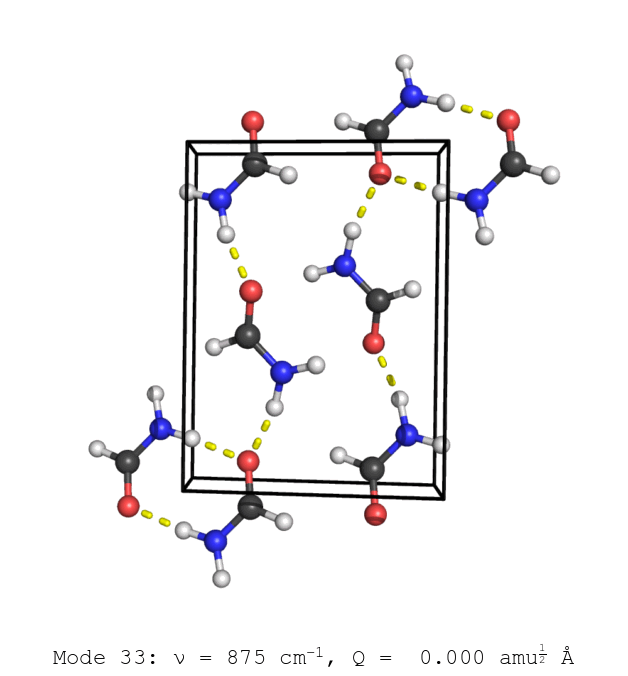

Supplement: Supplementary file 2 — ct3c00578_si_002.zip [file ct3c00578_si_002.zip › crystal_paper_gifs/PBED3_A_NHTWIST.gif]

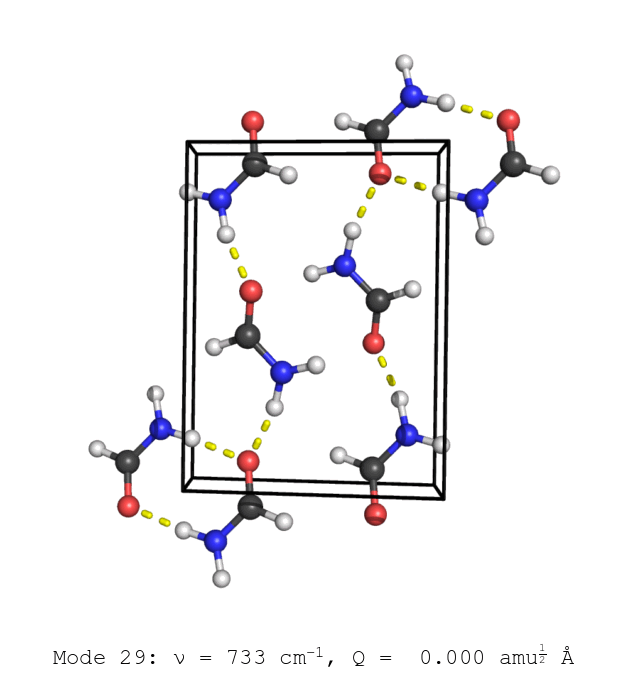

Supplement: Supplementary file 2 — ct3c00578_si_002.zip [file ct3c00578_si_002.zip › crystal_paper_gifs/PBED3_A_NHWAG.gif]

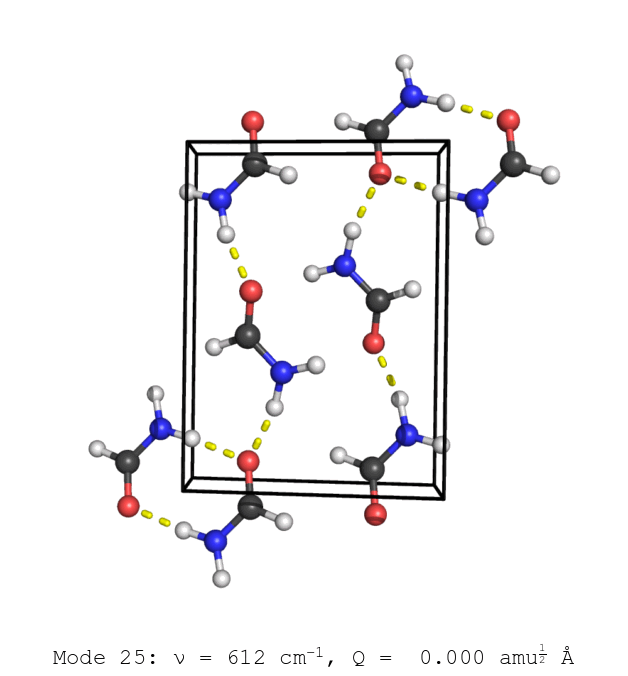

Supplement: Supplementary file 2 — ct3c00578_si_002.zip [file ct3c00578_si_002.zip › crystal_paper_gifs/PBED3_A_OCNBEND.gif]

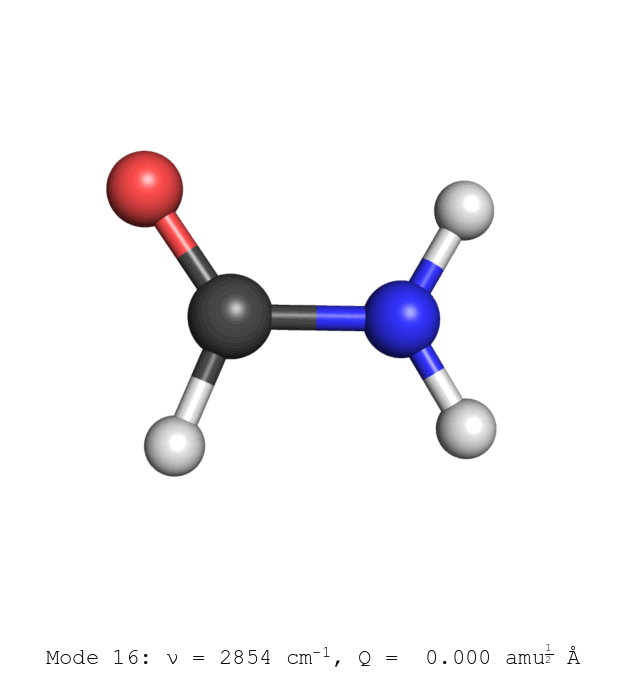

Supplement: Supplementary file 2 — ct3c00578_si_002.zip [file ct3c00578_si_002.zip › crystal_paper_gifs/PBED3_MON_CH.gif]

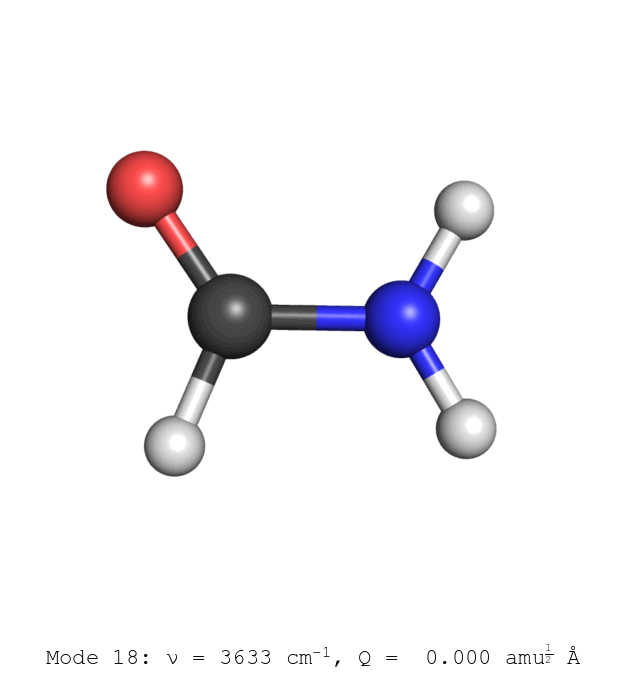

Supplement: Supplementary file 2 — ct3c00578_si_002.zip [file ct3c00578_si_002.zip › crystal_paper_gifs/PBED3_MON_NHASYMM.gif]

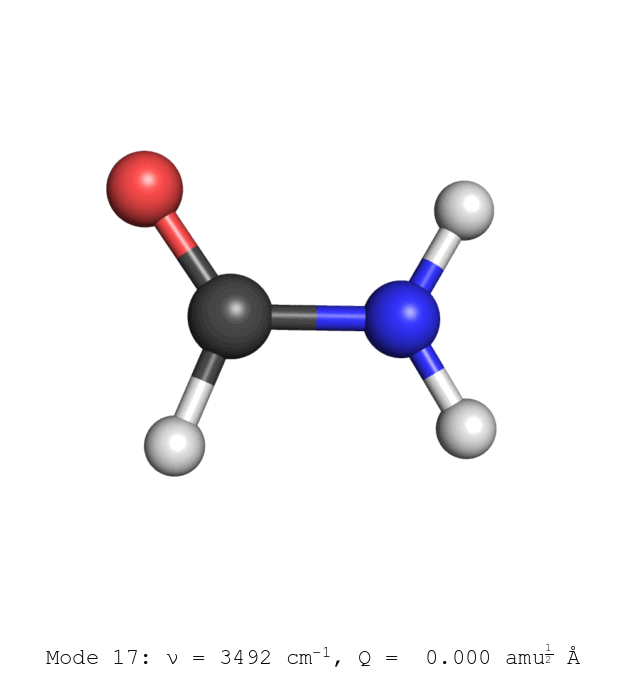

Supplement: Supplementary file 2 — ct3c00578_si_002.zip [file ct3c00578_si_002.zip › crystal_paper_gifs/PBED3_MON_NHSYMM.gif]

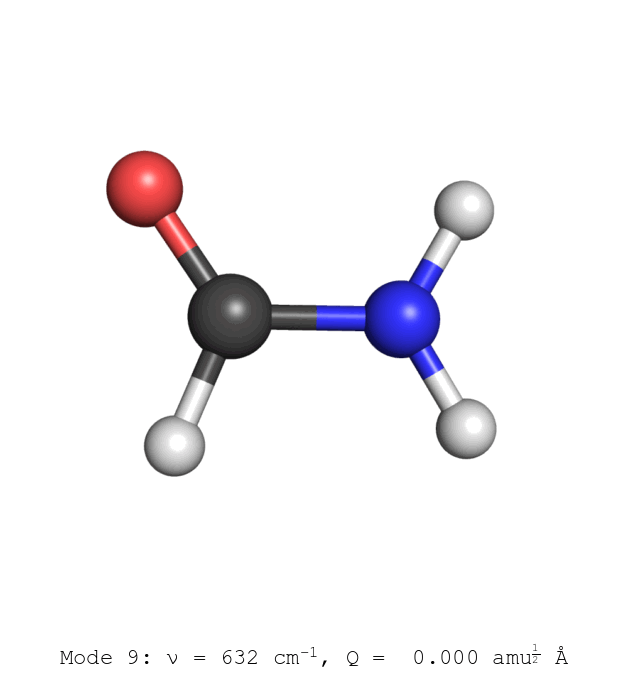

Supplement: Supplementary file 2 — ct3c00578_si_002.zip [file ct3c00578_si_002.zip › crystal_paper_gifs/PBED3_MON_NHTWIST.gif]

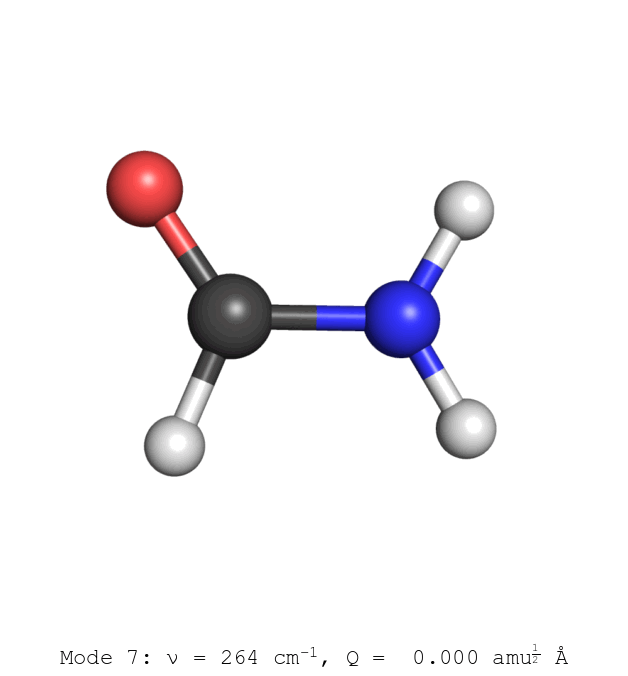

Supplement: Supplementary file 2 — ct3c00578_si_002.zip [file ct3c00578_si_002.zip › crystal_paper_gifs/PBED3_MON_NHWAG.gif]

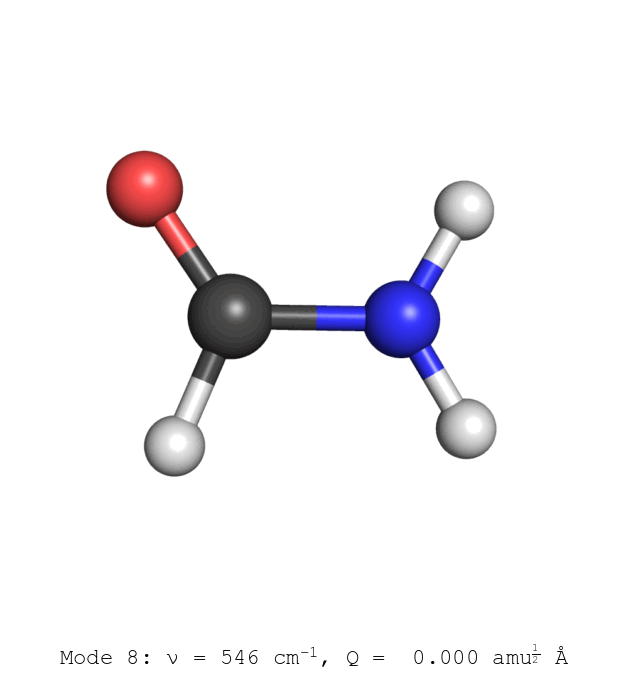

Supplement: Supplementary file 2 — ct3c00578_si_002.zip [file ct3c00578_si_002.zip › crystal_paper_gifs/PBED3_MON_OCNBEND.gif]
